# Synergistic Hepatoprotective Effects of Mesenchymal Stem Cells and Platelet-Rich Plasma in a Rat Model of Bile Duct Ligation-Induced Liver Cirrhosis

**DOI:** 10.3390/cells13050404

**Published:** 2024-02-26

**Authors:** Shivaraju Shivaramu, Swapan Kumar Maiti, Shajahan Amitha Banu, Elangovan Kalaiselvan, Khan Sharun, Mamta Mishra, Divya Mohan, Sangeetha Palakkara, Sunil Kumar, Monalisa Sahoo, Jürgen Hescheler

**Affiliations:** 1 Division of Surgery, ICAR-Indian Veterinary Research Institute, Izatnagar, Bareilly 243122, Uttar Pradesh, India; shivaraju558@gmail.com (S.S.); amianchu@gmail.com (S.A.B.); selvanholmes@gmail.com (E.K.); sharunkhansk@gmail.com (K.S.); mamtamishra652@gmail.com (M.M.); divyadhruvam@gmail.com (D.M.); sangeethapalakkara@gmail.com (S.P.); 2 Graduate Institute of Medicine, Yuan Ze University, Taoyuan 32003, Taiwan; 3 Division of Extension Education, ICAR-Indian Veterinary Research Institute, Izatnagar, Bareilly 243122, Uttar Pradesh, India; sunil.pearl27@gmail.com; 4 Division of Pathology, ICAR-Indian Veterinary Research Institute, Izatnagar, Bareilly 243122, Uttar Pradesh, India; vety.lisa@gmail.com; 5 Institute of Neurophysiology, University of Cologne, 50931 Cologne, Germany; j.hescheler@uni-koeln.de

**Keywords:** liver regeneration, liver cirrhosis, bile duct ligation, rat, adipose-derived mesenchymal stem cells

## Abstract

Liver cirrhosis poses a global health challenge marked by significant prevalence and mortality. Current therapeutic options are limited by high costs and immune-mediated rejection, necessitating the exploration of innovative strategies to enhance hepatic self-rehabilitation, and counteract the underlying pathological mechanisms. We evaluated the hepatoprotective activity of rat adipose-derived mesenchymal stem cells (ADMSCs) in combination with platelet-rich plasma (PRP) and recombinant human hepatocyte growth factor (rh-HGF) on a rat model of liver fibrosis/cirrhosis induced by bile duct ligation (BDL). Treatment with PRP or rh-HGF alone did not yield significant hepatoprotection in the BDL-induced liver cirrhosis model. However, ADMSC transplantation alone exhibited the potential to alleviate impaired liver conditions. The combination of PRP and rh-HGF demonstrated superior ameliorative effects compared to either treatment alone. Notably, the combination of ADMSC + PRP or ADMSC + rh-HGF significantly enhanced hepatoprotective capacity compared to individual or combined PRP and rh-HGF therapies. Injection of ADMSC via the tail vein reduced inflammation, hepatocyte damage, and collagen deposition, improving overall liver function. This improvement was more pronounced when ADMSC was administered with PRP and rh-HGF versus monotherapy. Our study concludes that ADMSCs exert antifibrotic effects by inhibiting hepatic stellate cell proliferation, collagen synthesis, and inducing apoptosis. ADMSCs also demonstrate immune-modulatory effects and transdifferentiate into hepatic progenitor cells, secreting trophic factors, cytokines, and chemokines that promote impaired liver regeneration. The observed arrest in liver fibrosis progression highlights the potential therapeutic impact of these interventions.

## 1. Introduction

The liver, a major organ, performs vital functions such as homeostasis, protein and glucose synthesis and storage, immune defense, and blood detoxification [1,2]. Disorders of liver cells lead to conditions such as cirrhosis, hepatitis, and hepatocellular carcinoma. Effective treatment protocols for these diseases are currently lacking. Hepatic injury results in failed regeneration due to excessive extracellular matrix (ECM) and collagen, leading to liver fibrosis [3,4]. Fibrosis progresses to cirrhosis with associated morbidity and mortality [5,6]. The end-stage of progressive fibrosis is cirrhosis, which can be distinguished by the development of scar rings, as well as a septum that encompasses the hepatocyte nodules. The accumulation of extracellular matrix occurs mainly due to decreased degradation and increased collagen synthesis [6].

Several efforts have been made to develop a therapy with low toxicity that contains anti-inflammatory, antioxidant, antiapoptotic and antifibrotic properties. These properties are integral in the prevention and treatment of fibrosis/cirrhosis. To overcome these hurdles, cell-based therapy and platelet-rich plasma (PRP) have been proposed as novel alternatives to liver transplantation. Mesenchymal stem cells (MSCs) act as an alternate source of cells for the repair of lesions apart from the primary hepatocytes in the hepatocyte transplant. This is because of the ability of MSCs to exhibit multiple and unlimited differentiation potentials. MSCs, when used as a potential therapeutic method, have special properties such as a self-renewal capability [7,8], strong in vitro proliferation [7,9] and have abundant sources for isolation of these cells [10,11]. Additionally, MSCs differentiate into multiple cell lineages, which give rise to daughter hepatocytes, and repopulate cells to improve liver function [1,12].

Undifferentiated adipose tissue-derived stem cells (ADMSCs) are considered an ideal agent for managing acute and critical liver conditions due to their easy availability and unrestricted propagation potential. Compared to bone marrow-MSCs and umbilical-MSCs, ADMSCs exhibit superior colony frequency and higher proliferation capacity [13]. PRP is an acceptable alternative therapy for tissue regeneration, releasing cytokines and growth factors stored in platelet alpha granules. These factors facilitate extracellular matrix remodelling, regulate angiogenesis, and influence stem cell proliferation, recruitment, and differentiation [14]. Hepatocyte growth factor (HGF), secreted by mesenchymal-derived cells, possesses diverse biological actions, including mitogenic, morphogenic, and antiapoptotic properties in various epithelial tissues, particularly hepatocytes. It plays a significant role in embryonic organogenesis, adult organ regeneration, and wound healing [15,16].

Transplantation of ADMSCs has shown therapeutic efficacy in rat models of carbon tetrachloride (CCl_4_)-mediated liver fibrosis [17]. The association between platelets, HGF, and hepatic fibrosis is supported by the findings of Takahashi et al. [18], who demonstrated that transfused human platelets improved CCl_4_-induced liver fibrosis in mice by increasing HGF levels in the liver, suppressing hepatic stellate cell (HSC) activation, inducing matrix metalloproteinase-9 expression, and inhibiting hepatocyte apoptosis. Consequently, HGF is considered not only to induce liver regeneration, but also to inhibit disease progression and ameliorate hepatic fibrosis in patients with intractable liver diseases [19]. Additionally, pretreatment of human ADMSCs with PRP and recombinant human hepatocyte growth factor (rh-HGF) has been found to enhance the efficacy of stem cell therapy in alleviating liver fibrosis in a mouse model [20]. However, the effects of ADMSCs, PRP, and rh-HGF on rat liver cirrhosis induced by obstructive cholestasis have been studied in relatively few trials. Although several studies have evaluated these therapeutic modalities as monotherapy, none have studied the impact of their combination. Therefore, the current research was conducted with the objectives of evaluating the hepatoprotective activity of rat ADMSCs in conjunction with PRP and rh-HGF on the liver fibrosis/cirrhosis model, and understanding the impact of their combination.

## 2. Materials and Methods

### 2.1. Experimental Animals

A total of 208 rats weighing around 250–300 g belonging to either sex (1:1) were utilised for this study. All rats received a standard diet, had unrestricted access to water, and followed a 12 h dark/light cycle. The animals were housed individually in cages under consistent management conditions throughout the study. A two-week acclimatisation period was implemented for all rats before the commencement of the study. Eight (8) male Wistar albino rats were used for the isolation, culture, and characterisation of ADMSCs. Twenty (20) male Albino Wister rats were used to prepare and characterise PRP. The bile duct ligation (BDL) experiment was conducted in 9 groups for 20 rats per group (*n* = 180). The animals were evaluated for a period of 42 days starting from the 7th day postsurgically.

### 2.2. Isolation, Culture, and Characterisation of ADMSCs

Male Wistar albino rats (*n* = 8, 250–300 g) were anesthetised using a xylazine (Xylaxin, Indian Immunologicals Ltd., Hyderabad, Telangana, India) and ketamine (Aneket, Neon Laboratories Ltd., Thane, Mumbai, India) combination. The abdominal region was aseptically prepared, and fat pads from subcutaneous deposits were collected from both sides of the abdomen. The adipose tissue was transported in Dulbecco’s phosphate-buffered saline (DPBS) (Catalog # 14190144, Gibco, Carlsbad, CA, USA) to the laboratory. The tissue was washed, minced, and then centrifuged in the lab at 2000 rpm for 5 min. The top fat layer was washed with DPBS, treated with 0.18% collagenase type-I (Catalog # 17100017, Gibco, Carlsbad, CA, USA), and agitated gently for 40 min at 37 °C. The enzyme activity was neutralised with complete media. The homogenised tissue was filtered and centrifuged at 1200 rpm for 10 min to collect the cell pellet. The pellet was resuspended in a complete medium and seeded in 25 cm^2^ cell culture flasks at 10^4^ cells/cm^2^. The adherent cells were incubated at 37 °C in a humidified atmosphere of 5% CO_2_ and 95% air. After 24–48 h, unattached cells were removed by washing with DPBS, and fresh media was added. When the flasks reached 70–80% confluence, cells were harvested (passaged). For differentiation studies and in vivo experiments, third passage (P3) cells were used. Mesenchymal progenitors were quantified through the colony forming unit-fibroblast (CFU-F) and cell viability assays. The ADMSCs were characterised by trilineage differentiation and urea and albumin secretion.

### 2.3. Hepatogenic Differentiation and Characterisation

A two-step procedure was used to achieve hepatic differentiation. The cells were grown in Dulbecco’s Modified Eagle Medium (Catalog # 11885084, Gibco, Carlsbad, CA, USA) supplemented with 10% fetal bovine serum (Catalog # 16140071, Gibco, Carlsbad, CA, USA), 20 ng/mL insulin-like growth factor 1 (IGF-1) (Catalog # I3769, Sigma-Aldrich, St. Louis, MO, USA), 20 ng/mL HGF (Catalog # H9661, Sigma-Aldrich, St. Louis, MO, USA), and 10^−7^ M/L dexamethasone (Catalog # A13449, Gibco, Carlsbad, CA, USA) for the first seven days. Oncostatin M (Catalog # PHC5015, Gibco, Carlsbad, CA, USA) was introduced to the stated medium at a concentration of 10 ng/mL in the second phase, which lasted until the 21st day of differentiation. Differentiated cells were incubated for 24 h in a medium containing 5 mmol/L NH_4_Cl at 37 °C in 5% CO_2_. Following incubation, the resulting supernatant was collected, and the quantity of urea was determined using a colourimetric test, as directed by the manufacturer (Catalog # MAK006, Sigma-Aldrich, St. Louis, MO, USA). The decrease of ammonia generated by urea hydrolysis is the basis for this test. As a negative control, undifferentiated MSCs were utilised. In addition, the concentration of albumin secreted into the culture medium on days 0, 7, 14, and 18 was measured using a calorimeter, as per the manufacturer’s instructions (Catalog # MAK124, Sigma-Aldrich, St. Louis, MO, USA).

### 2.4. Preparation and Characterisation of Platelet-Rich Plasma

Twenty male Albino Wistar rats served as blood donors. The rats were anesthetised with ketamine (50 mg/kg, intraperitoneal) and xylazine (5 mg/kg, intraperitoneal), and blood was drawn via closed heart puncture. Platelet, white blood cell (WBC), and red blood cell (RBC) counts were measured in both PRP and whole blood using a blood analyser. PRP characteristics were compared with whole blood parameters, and the factor increase in platelet concentration was calculated. Platelet dose in PRP, factor increase in platelet, WBC, and RBC concentration, and platelet capture efficiency (%) were determined. For a complete blood count, 1 mL of blood was collected from each animal in an ethylenediamine tetraacetic acid (EDTA) vial. The blood was centrifuged twice, and PRP was obtained following standard protocols [21,22]. PRP was frozen at −20 °C until use, and was activated with CaCl_2_ (0.8 mL PRP + 0.2 mL 10% CaCl_2_) before in vitro experiments [23]. Platelets were counted using an automated blood counter, with counts ranging from 307 K/µL to 745 K/µL. PRP was classified according to the PAW (platelets, activation, and WBC) classification system.

### 2.5. Surgical Techniques of Bile-Induced Cholestasis

The rats were anesthetised with xylazine (5 mg/kg) and ketamine (50 mg/kg) intraperitoneally. A midline laparotomy (approximately 2 cm) was performed, and the abdominal cavity was exposed. The common bile duct (CBD) was carefully separated from the portal vein and hepatic artery using microserrated forceps. Three ligatures were placed around the bile duct at specific joining points. The abdominal layers were sutured, and the skin was closed using nonabsorbable sutures. Postoperative antibiotic and anti-inflammatory medications were administered for 7 days.

### 2.6. Experimental Design

**Figure 1:**
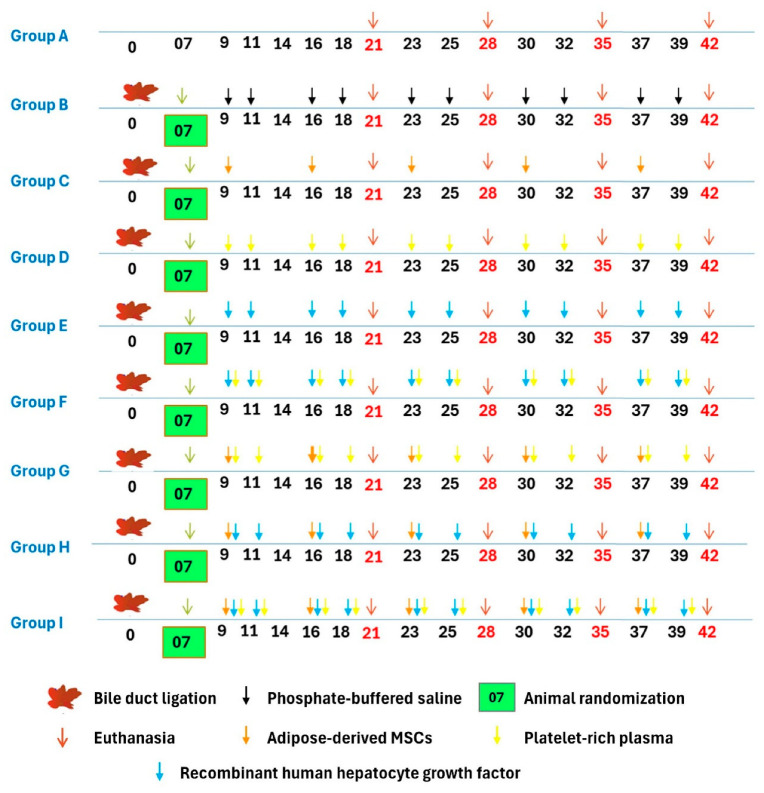
Graphical abstract of the experimental design showing the different groups and their corresponding treatment.

The BDL experimental animals were randomly divided after the 7th day of postsurgery into 8 groups, viz., Groups B, C, D, E, F, G, H, and I of 20 animals each, and Group A (sham control) animals underwent the separation of the common bile duct without ligation (Figure 1). The duration of the study was 42 (6 weeks) days with standard feeding and management conditions as per the Institutional Animal Ethic Committee norms. Group C, G, H, and I animals were given 1 × 10^6^ cells per week per animal intravenously (tail vein), (total dose of 5 × 10^6^ stem cells/rat). Group D, F, G, and I animals received 0.5 mL/kg CaCl_2_ activated PRP twice weekly from the 2nd week to the 6th week of postsurgery (2nd, 3rd, 4th, 5th, and 6th weeks) subcutaneously. Group E, F, H, and I animals were administered rh-HGF intravenously at the dose rate of 0.35 mg/kg twice weekly from the 2nd week of post-surgery (2nd, 3rd, 4th, 5th, and 6th weeks), and liver samples were collected for histopathology, immunohistochemistry, and tracking of transplanted cells.

### 2.7. Clinical Signs and Body Weight

The experimental rats were observed closely for the exhibition of clinical and behavioural abnormality, and their body weight was recorded at weekly intervals during the course of the study.

### 2.8. Biochemical Liver Function Analysis

A volume of 1 mL of blood was collected via periorbital plexus from rats at 0, 21st, 28th, 35th, and 42nd days, and serum was separated and stored at −20 °C. The biochemical parameters of aspartate aminotransferase (AST), alanine aminotransferase (ALT), total protein, albumin, and globulin gamma-glutamyl transferase (GGT), alkaline phosphatase (ALP), total bilirubin, and direct bilirubin were estimated by different methods by using commercially available standard kits. Serum matrix metalloproteinase-2 (MMP-2) was estimated by using an ELISA kit (Catalog # ITEH0017, ImmunoTag, St. Louis, MO, USA) at 0, 21st, 28th, 35th, and 42nd days/(0, 3rd, 4th, 5th, and 6th weeks. Tissue inhibitors of matrix metalloproteinases-2 (TIMP-2) and HGF were estimated using rat TIMP-2 ELISA kit (Catalog # ITER0065, ImmunoTag, St. Louis, MO, USA) and rat HGF ELISA kit (Catalog # ERA21RB, Invitrogen, Waltham, MA, USA) at 0, 21st, 28th, 35th, and 42nd days, respectively. The serum oxidative stress markers such as the Antioxidant Assay kit (Catalog # MAK334, Sigma-Aldrich, St. Louis, MO, USA) and the Superoxide Dismutase Assay Kit (SOD) (Catalog # 19160, Sigma-Aldrich, St. Louis, MO, USA) were used to assess oxidative stress indicators in serum samples from various treatment groups.

### 2.9. Morphology and Histopathological Examination

Gross hepatic morphology changes were observed in the animals. The left lateral lobe of the liver was fixed in 10% neutral buffered formalin (NBF). Sections were stained according to the hematoxylin and eosin staining protocol for microscopic evaluation of lesions. The area occupied by the portal spaces and the centrilobular venule was studied in all groups. Sections were also stained with Masson’s Trichrome to demonstrate collagen deposition in regenerated/degenerated liver tissues at different intervals of the experiment. Histopathological scoring of the hematoxylin and eosin (H&E) and Masson’s Trichrome stained sections were performed according to the Modified Histological Activity Index (HAI), score and the degree of liver fibrosis was scored using Knodell and METAVIR systems [24,25,26,27,28]. The fibrosis score was assessed on a five-point scale for cirrhosis. The activity score was graded according to the intensity of necro-inflammatory lesions.

### 2.10. Colour Digital Imaging

Colour photographs of the livers from different groups were captured at various time intervals using a handheld mobile camera. Digital imaging was employed to evaluate gross changes in liver size, shape, and other features, with subsequent analysis conducted using ImageJ software Version 1.41 (National Institutes of Health, Bethesda, MA, USA).

### 2.11. Immunohistochemistry (IHC)

Following anesthesia, liver tissues were excised and fixed overnight in 10% NBF. Subsequently, the tissues were transferred to fresh 10% NBF. Tissue sections (4–7 μm) were prepared on Poly-l-Lysine coated slides. Deparaffinisation involved xylene immersion, while rehydration used graded ethanol. Antigen unmasking was performed in citric buffer using microwave irradiation. Immunostaining steps included blocking nonspecific sites with 5% normal goat serum, washing, and titrating antibodies. Primary monoclonal antiproliferating cell nuclear antigen (PCNA) antibody (1:50 dilution) (Catalog # P8825, Sigma- Aldrich, St. Louis, MO, USA) was applied, and negative controls received diluent. Incubation occurred overnight at 4 °C. Following DPBS washing, biotinylated goat anti-mouse IgG (1:200) (Catalog # B7401, Sigma-Aldrich, St. Louis, MO, USA) was applied. The 3,3′-diaminobenzidine (DAB) substrate (Catalog # D7304, Sigma-Aldrich, St. Louis, MO, USA) provided colour to the sections. Counterstaining used Mayer’s hematoxylin, and was followed by rinsing and mounting.

### 2.12. Tracking of Transplanted Cells

When the ADMSCs reached 60–70% confluence in the third passage, 4′, 6-diamidino-2-phenylindole (DAPI; 1 μg/mL) (Catalog # D9542, Sigma-Aldrich, St. Louis, MO, USA) was introduced to the medium, and the cells were cultured for 12 h. Following incubation, the cells underwent six washes with DPBS, and the samples were examined using fluorescence microscopy. The DAPI-stained ADMSCs were trypsinised and counted, and approximately 2 × 10^6^ cells were intravenously injected through the tail vein. To identify DAPI + ADMSCs in the liver, tissue harvesting was performed on days 0, 5, and 9 after surgery, and frozen sections (5 μm thickness) were prepared and observed under a fluorescence microscope.

### 2.13. Statistical Analysis

The data were analysed using the Statistical Program for Social Sciences Version 20.0 software (IBM Corp, Armonk, NY, USA). One-way ANOVA was employed to compare means at various time intervals across different groups, while repeated measures of ANOVA were conducted to assess mean values within a group over different time intervals. The Tukey’s honest significant difference (HSD) post hoc test was employed following ANOVA analysis to ascertain any significant differences between the means of the groups. A significance level of *p* ˂ 0.05 (*) indicated statistical significance, and in some instances, *p* < 0.01 (**) was considered statistically significant. Graphs were generated using GraphPad Prism Version 7 (GraphPad Software Inc., La Jolla, CA, USA).

## 3. Result

### 3.1. Isolation, Culture, and Characterisation of ADMSCs

**Figure 2:**
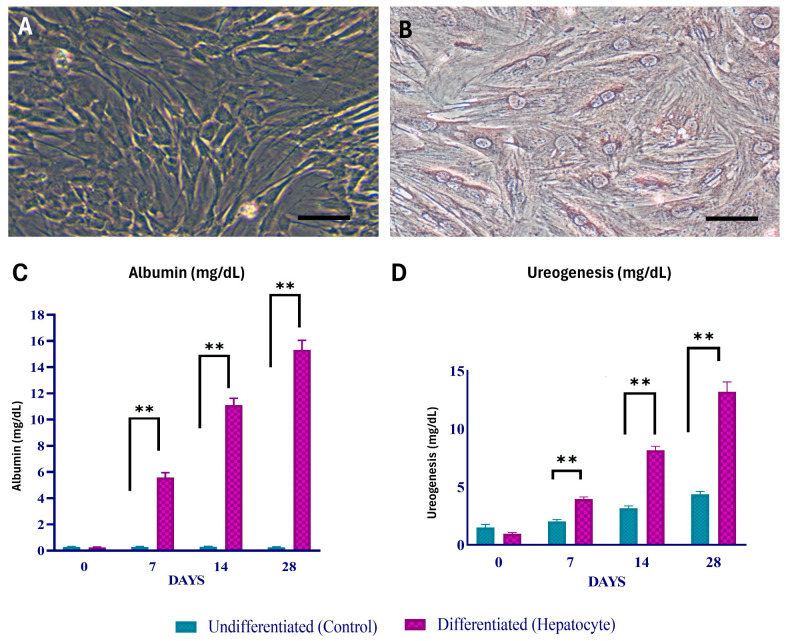
(**A**) ADMSCs in P3 stage. (**B**) Morphological features of the hepatogenic differentiated cells showing binucleated cells on day 21. Functional characteristics of hepatogenic differentiated cells based on albumin (**C**) and urea production (**D**). Scale bar—50 μm; ** indicates p-value less than 0.01.

The abdomen fat pads were harvested under aseptic conditions. Cell adhesion was observed in the primary culture after 2–3 days, with cells taking on a spindle-shaped structure and fibroblast shape by day 8. After two weeks, ADMSCs achieved 70–80% confluence. Third-passaged ADMSCs were used for differentiation and experiments. A colony-forming unit assay yielded 36.77 ± 7.3 fibroblast colonies. Cell viability significantly increased (*p* < 0.05) on day 7 and day 14 compared to day 0, with no significant (*p* > 0.05) difference between days 3, 7, and 14 in vitro conditions. Differentiation into osteoblasts, adipocytes, and chondrocytes was confirmed using specific staining methods. Under hepatogenic conditions, MSCs transitioned to polygonal hepatocyte morphology on the seventh day (Figure 2). Albumin production and ureagenesis significantly increased (*p* < 0.05) on days 7, 14, and 18 compared to undifferentiated cells (Figure 2).

### 3.2. Characterisation of PRP

PRP was prepared using a two-step gradient centrifugation technique. The PRP had substantially fewer RBCs and WBCs. The PRP used in this research falls within the PAW category P4-x-Bβ.

### 3.3. In Vivo Trial for Evaluation of the Hepatoprotective Effect of Rat ADMSC, PRP, and rh-HGF on Obstructive Cholestasis-Induced Liver Cirrhosis/Fibrosis

The three-point ligation obstructive cholestasis-induced liver fibrosis rat model was chosen for in vivo evaluation of the hepatoprotective effect of rat ADMSC, rh-HGF, and PRP individually and in combination based on the findings of biochemical, histology, and survival analysis.

### 3.4. Clinical Observations

Cholestasis-induced liver fibrosis in control BDL rats led to abnormal animal behaviour, severe diarrhea, epistaxis, rectal bleeding, dark-coloured urine, acholia, and ascites. Group B rats showed rough hair covering with undeveloped hairs around the surgical site. Physical activity was reduced in groups C, D, E, and F, but to a lesser extent than in the control group. Bleeding from the nostril and rectum occurred in some group B animals, whereas no bleeding was observed in the treatment groups. Group B animals exhibited nervous symptoms such as circling movements and tremors due to hepatic encephalopathy on certain days.

**Figure 3:**
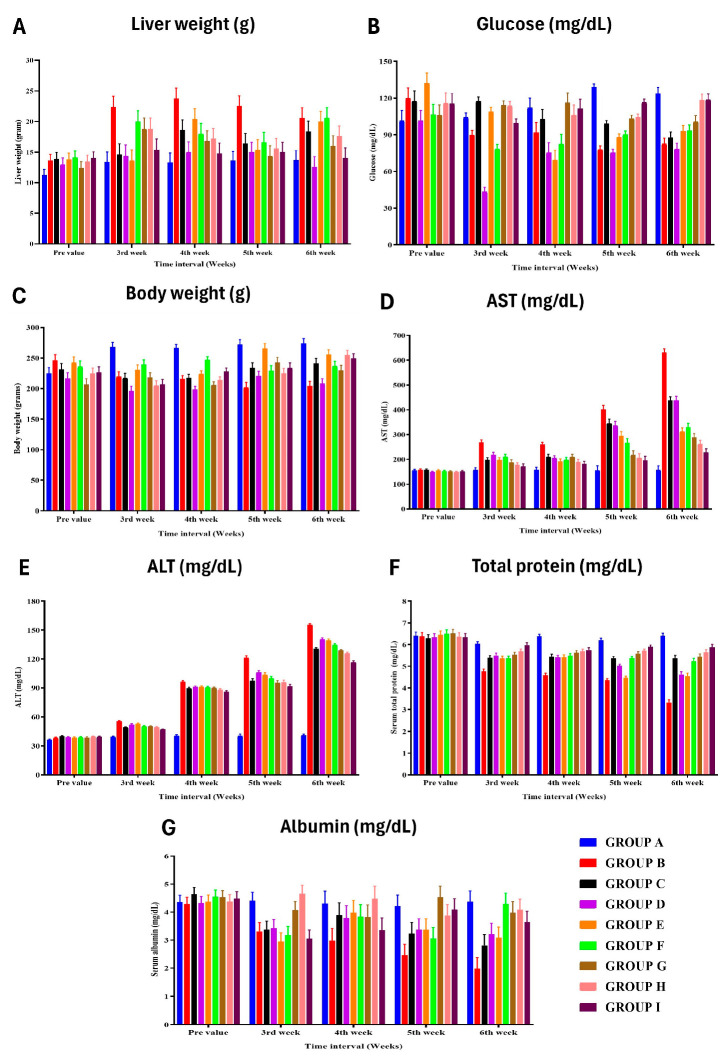
Effect of different treatment protocols on the (**A**) liver weight, (**B**) glucose, (**C**) body weight, (**D**) aspartate aminotransferase, (**E**) alanine aminotransferase, (**F**) total protein, and (**G**) albumin level of rats from different groups at various time intervals.

In the first weeks after surgery, all groups, including BDL control (group B), showed reduced body weight, although no group had a substantial reduction (Figure 3). Group A showed a significant increase in body weight during the third, fourth, fifth, and sixth weeks after surgery (Supplementary Table S1). However, group B experienced substantial weight loss in the fifth and sixth weeks. Animals treated with PRP alone (group D) demonstrated a reduction in body weight throughout the research period.

Group B showed an enlarged liver, while group D showed a reduced liver size (Supplementary Table S2). Groups G and H initially experienced hepatomegaly but recovered to nearly normal liver weight by the end of the study period (Figure 3). Group G showed increased liver weight at week 3, and group H showed increased liver weight at weeks 3 and 4. Group I animal’s maintained normal liver weight throughout the study. Appetite loss, reduction in oral food intake, weight loss, and reduced glycogen synthesis were all seen in group B. The body weight loss and glucose deficiency in rats treated with ADMSC/PRP, ADMSC/rh-HGF, and ADMSC/PRP/rh-HGF (group G, H, and I) were less.

### 3.5. Biochemical Liver Function Analysis

**Figure 4:**
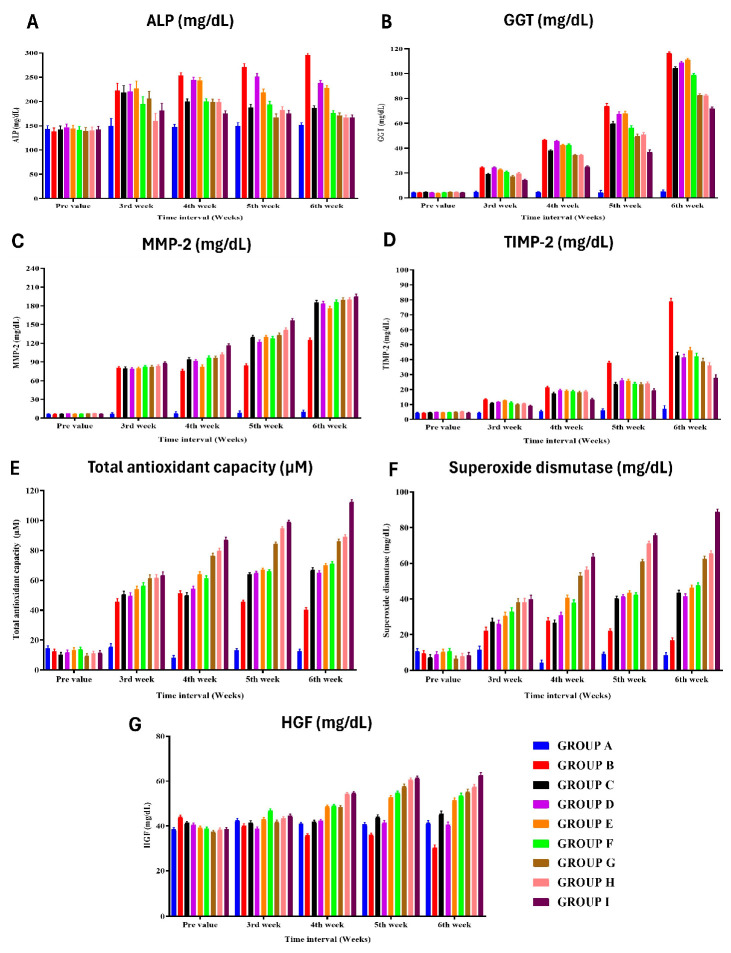
Effect of different treatment protocols on the (**A**) alkaline phosphatase, (**B**) gamma-glutamyl transferase, (**C**) matrix metalloproteinase-2, (**D**) tissue inhibitor of metalloproteinases 2, (**E**) total antioxidant capacity, (**F**) superoxide dismutase, and (**G**) serum hepatocyte growth factor level of rats from different groups at various time intervals.

The levels of AST and ALT in group B (control) showed increased values at various time points, indicating liver damage and proliferation (Figure 3) (Supplementary Tables S4 and S5). The highest AST value in group B was observed in the fifth week after surgery, while the highest ALT value was found in the fifth week and sixth week after surgery. Other treatment groups (C, D, E, F) showed lower AST and ALT values compared to group B. The levels of total protein, albumin, and globulin in group A had significantly higher serum total protein in the fifth week after surgery compared to group B (Supplementary Tables S6–S8). Group B had lower serum total protein compared to other groups, except group E. Group B also had lower serum albumin in the fifth and sixth weeks after surgery compared to some groups (G, F), while group D showed a reduction in albumin throughout the research period. Group B had higher levels of serum cholestasis markers such as ALP, GGT, total bilirubin, and direct bilirubin in various weeks after surgery compared to other groups (Supplementary Tables S9–S13). Group H had the least value of GGT in the third week after surgery, while group I had the highest HGF value. Biochemical lipid profile analysis revealed group B as having the highest total lipid and triglyceride values in various weeks after surgery compared to other groups (Supplementary Tables S14 and S15). HDL levels were higher in group I, while low-density lipoprotein (LDL) and total cholesterol levels were higher in group B compared to other groups (Supplementary Tables S17 and S18). The HGF and MMP-2 levels in Group B had higher levels of HGF and MMP-2 compared to some groups (Figure 4) (Supplementary Tables S22 and S23). Group I had the highest HGF and MMP-2 values in various weeks after surgery. TIMP-2 and oxidative stress (TAC) study in group B had the highest TIMP-2 values in the fifth and sixth weeks after surgery (Supplementary Table S24). TAC and superoxide dismutase levels were highest in group I, while group A had lower TAC and SOD values compared to group B (Figure 4) (Supplementary Tables S25 and S26).

### 3.6. Histopathological Observations

**Figure 5:**
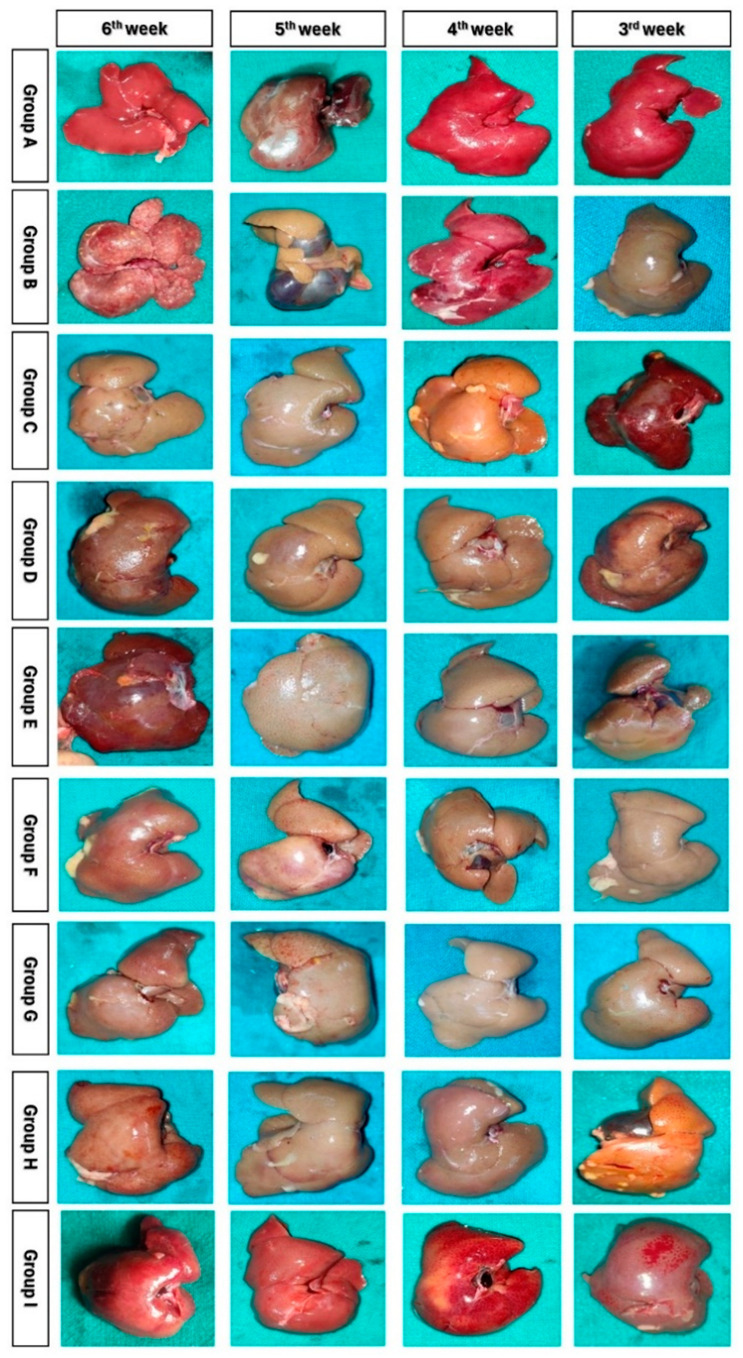
The colour images depict the gross appearance of the dorsal surface of the rat livers from Groups A–I at various time intervals.

**Figure 6:**
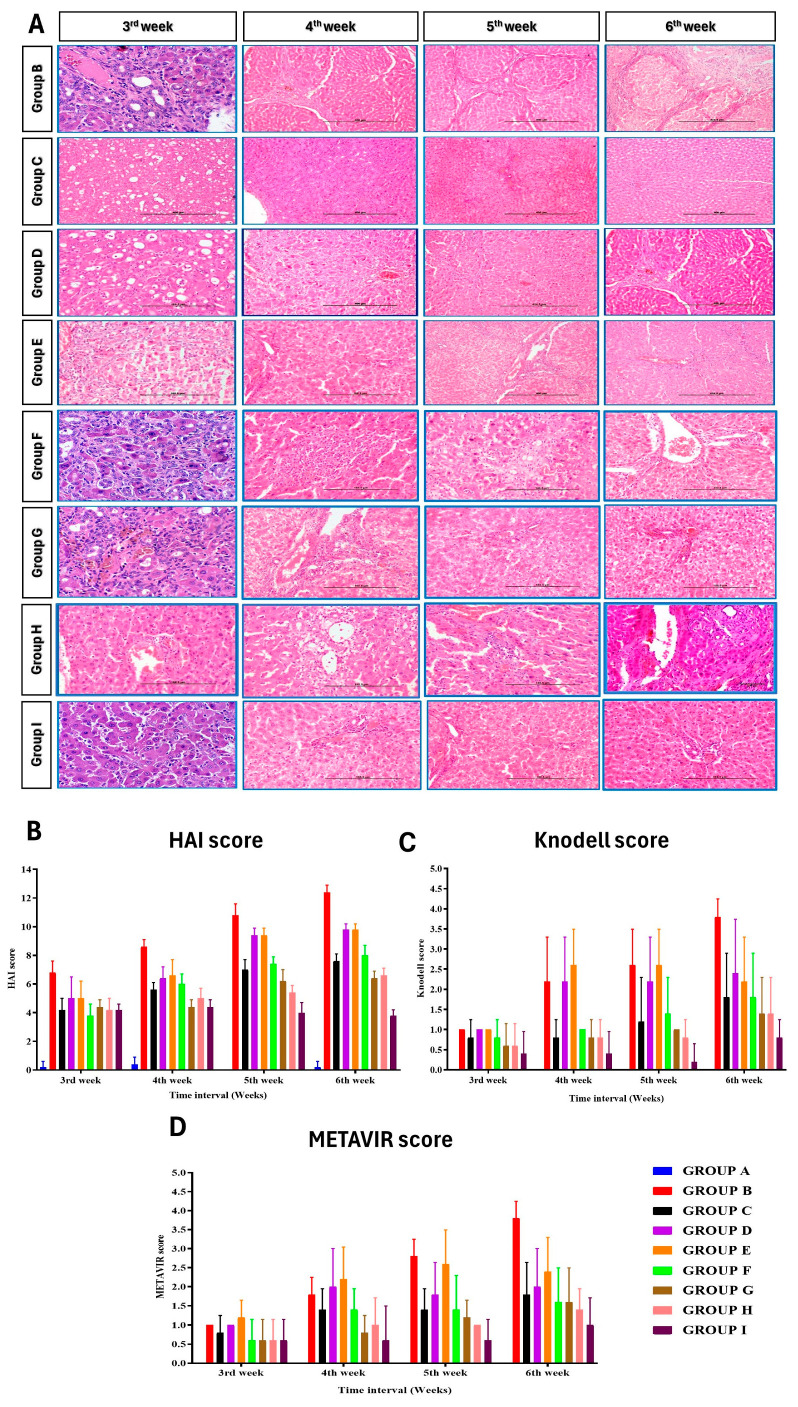
(**A**) Histological features of the liver from different groups throughout the study period at different time intervals (H&E). Histological evaluation based on the Modified Histological Activity Index—HAI (**B**), Knodell (**C**), and METAVIR (**D**) scoring systems. Scale bar—400 μm.

**Figure 7:**
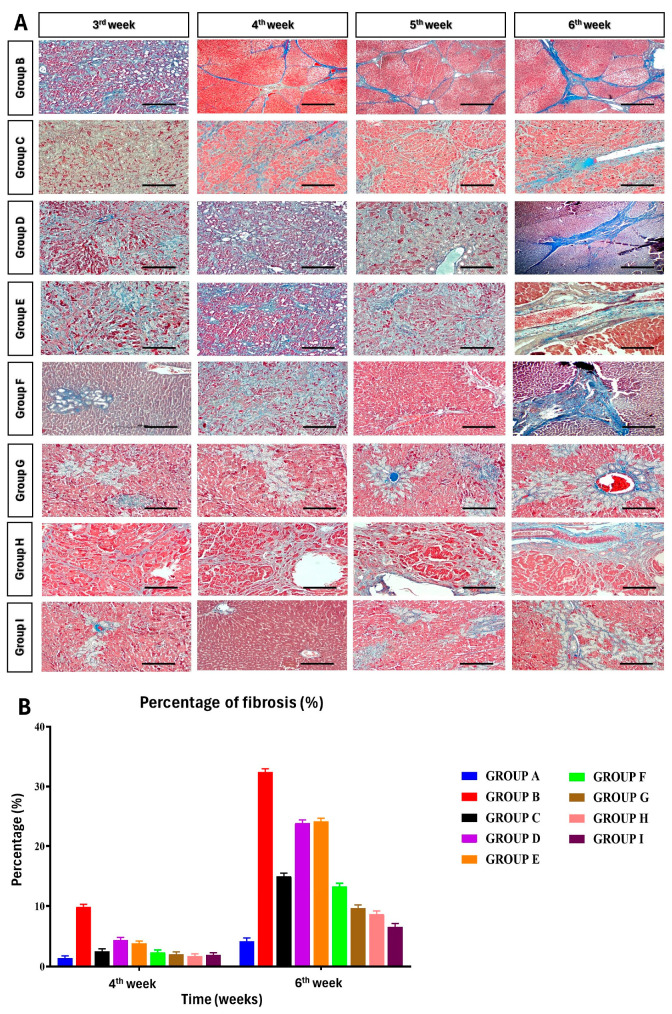
(**A**) Histological features of the liver from different groups throughout the study period at different time intervals (Masson trichrome staining). (**B**) Group I (ADMSC + rh-HGF + PRP) exhibited the highest decrease in fibrosis percentage reduction with 79.71 percent, followed by groups H (73.23 percent), G (70.25 percent), F (59.42 percent), C (53.86), D (26.86 percent), and E (25.50 percent). Scale bar—200 μm.

Throughout the study, group A showed normal liver histology, while group B exhibited liver damage and biliary cirrhosis (Figure 5). Group C (ADMSC-treated) preserved hepatic architecture, and group D (PRP-treated) showed regenerative potential. Group E (rh-HGF-treated) and group F (HGF + PRP-treated) demonstrated regenerative effects with minimal inflammation. Group H (ADMSC + rh-HGF-treated) showed anti-fibrotic potential. Group I (ADMSC + PRP + rh-HGF-treated) displayed hepatoprotective efficacy. Masson’s Trichrome staining confirmed increased collagen deposition in group B, followed by other treatment groups in descending order (Figure 6 and Figure 7). HAI score increased significantly at the 5th and 6th weeks after surgery in groups B, C, D, E, F, and G, but only at the 6th week after surgery in group I (Figure 6) (Supplementary Table S28). The Knodell score increased significantly (*p* < 0.05) at the end of the sixth week in groups B and E when compared to its baseline value, whereas other groups showed no significant variations within themselves (Figure 6) (Supplementary Table S28). Group B had the significantly (*p* < 0.05) highest Knodell score in the sixth week after surgery, followed by groups D, E, F, G, H, and I, and the significantly (*p* < 0.05) lowest in group A. However, groups D and C did not show any significant variations compared to group B. The METAVIR score increased significantly (*p* < 0.05) at the 4th, 5th, and 6th weeks in group B when compared to its baseline value prior to surgery, but there were no significant differences in the other groups (Supplementary Table S28). Group B had the significantly (*p* < 0.05) highest METAVIR score in the 5th week after surgery compared to all other groups, except for groups E, C, and F, with which it did not show any significant variations. When compared to the other groups, group B had the significantly (*p* < 0.05) highest METAVIR score in the sixth week after surgery. However, group I exhibited a significantly (*p* < 0.05) lower METAVIR score among the treatment groups when compared with group B (Figure 6).

### 3.7. Percentage of Fibrosis

Masson’s Trichrome stained the collagen fibers blue and the cell nuclei black. In group B BDL rats, the collagen fibers were thick and the mean percentage of fibrosis was assessed digitally, and at 4th and 6th weeks postsurgery, it was 9.9 percent and 32.43 percent correspondingly (Figure 7) (Supplementary Table S27).

### 3.8. Immunohistochemistry

**Figure 8:**
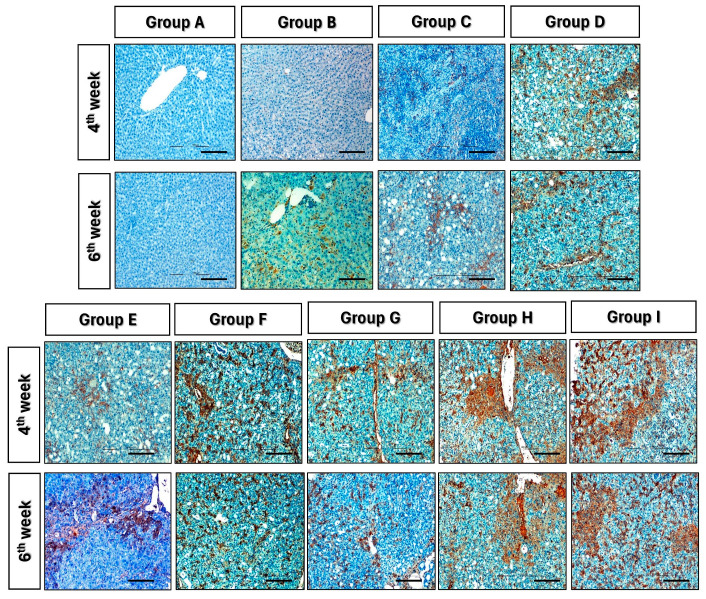
The PCNA expression in liver tissue samples evaluated by immunohistochemical staining in the 4th and 6th weeks. Scale bar—200 μm.

The PCNA expression in liver tissue was evaluated by immune histochemical staining with a monoclonal antibody to PCNA in the 4th and 6th weeks following surgery in various treatment groups. The PCNA-positive cells coloured dark yellow in the nucleus and showed a thin granular texture. On the third week, after ADMSC, PRP, and HGF transplantation in groups C, D, and E, there was a substantial increase in PCNA expression in the treatment groups compared to group B (control group) (Figure 8).

### 3.9. Tracking of Transplanted Cells

**Figure 9:**
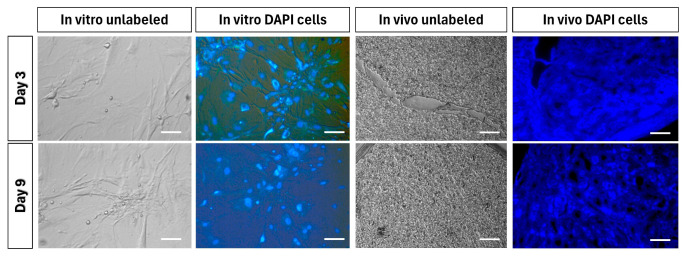
Tracking of transplanted DAPI-stained ADMSCs in the liver. Intravenously transplanted DAPI-stained ADMSCs were detected in the liver sections of BDL rats after three and nine days, with a higher number of cells observed after three days. Scale bar—100 μm.

In the third passage, DAPI staining was performed on ADMSCs in vitro, and the cell nuclei were observed to fluoresce under a fluorescent microscope. Intravenously transplanted DAPI-stained ADMSCs were detected in the liver sections of BDL rats after three and nine days, with a higher number of cells observed after three days. Unstained ADMSCs (control) did not produce fluorescence in both in vitro and in vivo experiments (Figure 9).

## 4. Discussion

Cholestatic fibrosis in rats can be effectively induced through a variety of chemical or surgical hepatic injuries. These include exposure to hepatotoxins such as alpha-naphthyl-isothiocyanate (ANIT), CCl_4_, and 3,5-diethoxycarbonyl-1,4-dihydrocollidine (DDC), as well as common bile duct ligation [29,30,31,32]. Recent studies have emphasized the importance of various surgical methods in generating extrahepatic cholestasis, particularly in rats [33,34] and mice [35]. However, the model of BDL for persistent cholestasis-induced liver cirrhosis remains the gold standard [35]. The BDL rat model of liver fibrosis triggers the proliferation of biliary epithelial cells (BECs) or oval cells, resulting in bile ductule proliferation, portal inflammation, fibrosis, and secondary biliary cirrhosis, closely resembling the pathology observed in humans [36]. Unlike other liver fibrosis models, which undergo self-regeneration and reversal, the BDL model does not exhibit spontaneous recovery from fibrosis [37,38]. We performed surgical ligation of the bile ducts using a three-point ligation technique on day 0, which induces progressive, nonreversible cholestasis leading to liver cirrhosis. Treatment was initiated once all animals exhibited clear clinical signs of cholestasis, which typically occurred by day 7. Subsequently, we compared the treatment groups with a control group of animals that had developed complete liver cirrhosis/fibrosis by the end of the study period. This comparison allowed us to assess the impact of the treatment interventions.

Each year, liver disease claims 3.5% of the global population [27]. Fibrosis, resulting from chronic and repeated liver injury, is the most common complication [39]. Hepatic fibrosis, triggered by various stimuli, involves similar molecular processes. Chronic damage such as cholestasis, caused by reduced bile flow, affects hepatocytes and the endothelial barrier, leading to increased collagen and ECM production by activated HSCs. Previous studies have shown that ADMSCs protect against acute or chronic liver failure and fibrosis [40]. HGF, a well-known antifibrotic cytokine, suppresses fibrogenic cytokine expression (transforming growth factor-β1 and platelet-derived growth factor-BB) and inhibits HSC proliferation and activation [41,42,43].

The current study found that MSCs obtained from adipose tissue adhered to plates after expansion in culture, in line with earlier reports [44]. The mitogenic factors promoting CFU proliferation, including epidermal growth factor, platelet-derived growth factor, basic fibroblast growth factor, insulin-like growth factor-1, and transforming growth factor-β, are not fully understood [45]. The primary functional tests for hepatocytes, urea, and albumin synthesis were successfully demonstrated in rat ADMSCs, confirming their typical hepatocyte morphological and functional characteristics in vivo, as reported by Ayatollahi et al. [46]. In the treatment protocol using PRP, platelet activation is a critical stage that affects tissue repair by releasing growth factors from platelet alpha granules [47]. To activate PRP, various procedures often involve the addition of autologous thrombin, calcium chloride (CaCl_2_), and collagen type-I [48]. The antifibrotic impact of 10% calcium chloride-activated PRP was examined in BDL rats in the current study. Previous investigations using 10% calcium chloride-activated PRP in a liver fibrosis model showed that growth factors are mainly released during the first week after administration [23,49]. Thus, BDL rats were treated twice weekly in this study to maximise the release of growth factors from PRP.

DeLong et al. [50] introduced the platelet, activation, WBC (PAW) grading system, based on platelet quantity, activation form, and presence of white cells. The PRP used in this research belongs to PAW category P4-x-Bβ. Martin-Sole et al. [51] obtained similar results with PRP in a rat model of renal ischemia-reperfusion, emphasising the importance of accurate quantification of cellular components and activator dosage in PRP studies.

In the current study, BDL control rats had significantly higher deaths due to major bleeding compared to sham control and other treatment groups, consistent with Akimoto et al. [52]. Rats treated with ADMSCs, ADMSCs/rh-HGF, ADMSCs/PRP, and ADMSCs/rh-HGF/PRP showed protection against cytotoxic bile acids, as evidenced by a lack of ruffled fur, circular movement, and gastrointestinal bleeding. The highest retention of normal hepatocytes was observed in groups I, G, and H. The weight loss and hepatomegaly in group B BDL rats may result from the detrimental impact of accumulated bile acids and soluble bile salts on hepatocytes over the experiment duration. Rats treated with ADMSC/PRP, ADMSC/rh-HGF, and ADMSC/PRP/rh-HGF (groups G, H, and I) showed prevention of weight loss and glucose deficiency, likely attributed to improved glucose and insulin homeostasis, in line with the study by Hao et al. [53] on diabetic rats, which highlighted the importance of multiple MSC transplantations for effective restoration and maintenance.

The effective hepatogenic differentiation of rat ADMSCs utilising HGF and IGF-1 demonstrates HGF’s role in liver regeneration, leading to a substantial reduction in AST and ALT levels compared to ADMSCs and HGF alone. The rise in blood HGF levels in groups E, H, and I after exogenous injection demonstrates the synergistic impact of stem cells and HGF. Combining HGF and VEGF in PRP promotes synergistic healing after hepatic resection, enhancing liver cell activity and regeneration [54]. Similar results were found in groups treated with PRP + ADMSC. Hepatic mesenchymal cells secrete HGF, reducing chronic inflammation and healing fibrosis [55]. These findings may explain the decrease in fibrosis in the groups treated with ADMSC in the current research. ADMSC, ADMSC + rh-HGF, ADMSC + PRP, and ADMSC + PRP + rh-HGF have all shown hepatoprotective effects, with no substantial difference in albumin and total protein levels between the BDL groups and the sham control group.

Mice treated with ADMSC pretreated with PRP and HGF showed reduced ALT levels, consistent with prior studies [20]. ADMSC transplantation, with or without rh-HGF/PRP, decreased ALT levels and improved lipid metabolism in BDL rats. HGF’s immunomodulatory effect, along with ADMSC, may contribute to hepatoprotection [56]. ADMSC + rh-HGF and ADMSC + PRP reduced fibrosis, and collagen levels in liver tissue decreased after ADMSC + rh-HGF, ADMSC + PRP, PRP + rh-HGF, and ADMSC + PRP + rh-HGF transplantation. ADMSC, PRP, and rh-HGF individually or in combination reduced fibrosis and collagen content in liver tissue. This study demonstrated improved efficacy of ADMSC + PRP + rh-HGF in BDL rats, protecting the liver from bile acid/salt-induced damage. Platelets in the liver secrete HGF, IGF-1, and VEGF, stimulating liver regeneration [57]. ADMSC + HGF + PRP reduced liver damage and fibrosis in CCL_4_-treated mice [20]. ADMSC pretreated with HGF and PRP inhibited fibrosis-related genes in vitro and in vivo [20]. This study suggests that ADMSC, PRP, and HGF alone or in combination reduce fibrosis and promote liver regeneration.

Our findings indicate that the intravenous injection of ADMSCs demonstrates a reduction in inflammation, hepatocyte damage, and fibrosis, leading to overall improvement in liver function. Enhanced hepatic metabolism is observed when ADMSCs are administered in conjunction with PRP and rh-HGF compared to when given alone. Despite showcasing an apparent halt in the progression of liver fibrosis, this study did not observe a reversal of bile duct proliferation. The precise mechanisms underlying ADMSC-mediated hepatic repair and fibrosis reduction remain to be fully elucidated.

An intriguing aspect to consider is the potential immune-mediated pseudotoxic effects induced by rh-HGF in normal rat kidneys, particularly with repeated doses [58]. An alternative approach to mitigate this concern involves utilising rat-HGF, which has been associated with reduced deposition of glomerular IgG [58]. However, our study opted for rh-HGF due to its common usage in hepatic regeneration studies, and its availability in our institute at the time of the study [59,60,61]. Future research endeavors could explore whether the utilisation of rat-HGF might enhance the regenerative potential of ADMSC and PRP compared to rh-HGF. This investigation could provide valuable insights into optimizing therapeutic strategies for hepatic regeneration while minimizing potential adverse effects.

## 5. Conclusions

The present study concluded that therapy with PRP or rh-HGF alone did not result in substantial hepatoprotection in the rat BDL model of liver cirrhosis. The combination of PRP and rh-HGF, on the other hand, showed somewhat greater ameliorative benefits than either PRP or rh-HGF alone. Furthermore, this study found that ADMSCs were easily accessible and extendable. ADMSC transplantation through the tail vein migrates to damaged liver tissue, develops into hepatocyte-like cells, decreases inflammation, decreases fibrosis, and has antioxidant effects on liver fibrosis. The results of this study showed the synergistic ameliorative and regeneration ability of ADMSCs, PRP, and HGF on cholestasis-induced liver fibrosis/cirrhosis, which have the potential to be put into practice.

## Data Availability

The original contributions presented in this study are included in the article/Supplementary Material; further inquiries can be directed to the corresponding author.

## Supplementary Materials

The following supporting information can be downloaded at: https://www.mdpi.com/article/10.3390/cells13050404/s1. Table S1: Effect of different treatment protocols on the body weight of BDL rats at various time intervals. Table S2: Effect of different treatment protocols on the liver weight of BDL rats at various time intervals. Table S3: Effect of different treatment protocols on the serum glucose of BDL rats at various time intervals. Table S4: Effect of different treatment protocols on the aspartate aminotransferase (AST) of BDL rats at various time intervals. Table S5: Effect of different treatment protocols on the alanine aminotransferase (ALT) of BDL rats at various time intervals. Table S6: Effect of different treatment protocols on the serum total protein of BDL rats at various time intervals. Table S7: Effect of different treatment protocols on the serum albumin of BDL rats at various time intervals. Table S8: Effect of different treatment protocols on the serum globulin of BDL rats at various time intervals. Table S9: Effect of different treatment protocols on the alkaline phosphatase of BDL rats at various time intervals. Table S10: Effect of different treatment protocols on the gamma glutamyl transferase of BDL rats at various time intervals. Table S11: Effect of different treatment protocols on the total bilirubin of BDL rats at various time intervals. Table S12: Effect of different treatment protocols on the direct bilirubin of BDL rats at various time intervals. Table S13: Effect of different treatment protocols on the indirect bilirubin of BDL rats at various time intervals. Table S14: Effect of different treatment protocols on the total lipids of BDL rats at various time intervals. Table S15: Effect of different treatment protocols on the triglycerides of BDL rats at various time intervals. Table S16: Effect of different treatment protocols on the high-density lipoprotein (HDL) of BDL rats at various time intervals. Table S17: Effect of different treatment protocols on the low-density lipoprotein (LDL) of BDL rats at various time intervals. Table S18: Effect of different treatment protocols on the total cholesterol of BDL rats at various time intervals. Table S19: Effect of different treatment protocols on the serum creatinine of BDL rats at various time intervals. Table S20: Effect of different treatment protocols on the serum urea of BDL rats at various time intervals. Table S21: Effect of different treatment protocols on the blood urea nitrogen (BUN) of BDL rats at various time intervals. Table S22: Effect of different treatment protocols on the serum hepatocyte growth factor level of BDL rats at various time intervals. Table S23: Effect of different treatment protocols on the MMP-2 of BDL rats at various time intervals. Table S24: Effect of different treatment protocols on the TIMP-2 of BDL rats at various time intervals. Table S25: Effect of different treatment protocols on the total antioxidant capacity (TAC) of BDL rats at various time intervals. Table S26: Effect of different treatment protocols on the superoxide dismutase (SOD) of BDL rats at various time intervals. Table S27: Effect of different treatment protocols on the percentage of fibrosis of BDL rats at various time intervals. Table S28: Effects of different treatment protocol on necro-inflammatory and fibrosis scores (mean±SD) in the BDL rats.
