# Supplementary material for: Synergistic Hepatoprotective Effects of Mesenchymal Stem Cells and Platelet-Rich Plasma in a Rat Model of Bile Duct Ligation-Induced Liver Cirrhosis
Source: Cells. 2024 Feb 26;13(5):404. doi: 10.3390/cells13050404 (PMC10931218; doi:10.3390/cells13050404)
Supplement: Supplementary file 1 [file cells-13-00404-s001.zip › cells-2846195-supplementary.pdf]

Supplementary files

Supplementary Tables

Table 1: Effect of different treatment protocols on the body weight of BDL rats at various time intervals.

	Pre value	3rd week	4th week	5th week	6th week
Group A	225±9.34	268±7.78^{ac*}	266.6±5.46^{a*}	272±8.23^{a*}	274±7.83^{a*}
Group B	246.4±9.34	219.4±7.78^{bd}	215.2±5.46^{b}	202.00±8.23^{b*}	204.0±7.83^{b*}
Group C	231.8±9.34	217.4±7.78^{bc}	217.8±5.46^{b}	234.2±8.23^{ab}	241.4±7.83^{ac}
Group D	216.6±9.34	196±7.78^{b}	198.4±5.46^{b}	220.4±8.23^{b}	208.4±7.83^{b}
Group E	242.6±9.34	230.8±7.78^{abc}	224±5.46^{c}	265.6±8.23^{ac}	255.8±7.83^{ad}
Group F	235.8±9.34	239.2±7.78^{cd}	247±5.46^{ac}	229.2±8.23^{bc}	236.8±7.83^{ac}
Group G	206.8±9.34	218.2±7.78^{bd}	205.8±5.46^{b}	242.8±8.23^{ab}	230.2±7.83^{cd}
Group H	224.4±9.34	205±7.78^{bd}	214±5.46^{b}	224.8±8.23^{b}	254.8±7.83^{ad}
Group I	226.4±9.34	207±7.78^{bd}	228±5.46^{c}	234.8±8.23^{ab}	249.4±7.83^{ad}

Values are given as mean±SE for 5 rats in each interval in each group (n=20).
Each value not sharing a common letter superscript is significantly different (P<0.05).
* Mean values differ significantly within the group (P<0.05).

Table 2: Effect of different treatment protocols on the liver weight of BDL rats at various time intervals.

	Pre value	3rd week	4th week	5th week	6th week
Group A	11.25±0.96	13.4±1.62^{a}	13.33±1.54^{a}	13.6±1.5^{a}	13.7±1.54
Group B	13.61±1.05	22.38±1.77^{b*}	23.76±1.69^{b}	22.57±1.64^{b}	20.57±1.68*
Group C	13.91±1.05	14.6±1.77	18.6±1.69*	16.4±1.64	18.4±1.68
Group D	13±1.05	14.4±1.77	15±1.69^{ac}	15±1.64	12.6±1.68
Group E	13.79±1.05	13.6±1.77^{ac}	20.4±1.69*	15.4±1.64	20±1.68*
Group F	14.15±1.05	20±1.77	18±1.69	16.6±1.64	20.6±1.68*
Group G	12.41±1.05	18.8±1.77*	16.8±1.69^{ac*}	14.4±1.64	16±1.68
Group H	13.46±1.05	18.8±1.77*	17.2±1.69	15.6±1.64	17.6±1.68
Group I	14±1.05	15.4±1.77^{ac}	14.8±1.69	15±1.64	14±1.68

Values are given as mean±SE for 5 rats in each interval in each group (n=20).
Each value not sharing a common letter superscript is significantly different (P<0.05).
* Mean values differ significantly within the group (P<0.05).

Table 3: Effect of different treatment protocols on the serum glucose of BDL rats at various time intervals.

	Pre value	3rd week	4th week	5th week	6th week
Group A	101.6±8.28	104.4±3.52^{ac}	112±8.03^{ac}	129±2.66^{a}	124±4.64^{a}
Group B	120±8.28	90.0±3.52^{ad}	92±8.03^{abc}	78.2±2.66^{bd}	82.6±4.64^{b}
Group C	117.6±8.28	117.4±3.52^{c}	102.8±8.03^{abc}	99±2.66^{cef}	87.6±4.64^{b}
Group D	101.6±8.28	43.6±3.52^{b}	75.6±8.03^{ab}	75.6±2.66^{b}	78.6±4.64^{b}
Group E	132.2±8.28	109±3.52^{ce}	69.4±8.03^{b}	88±2.66^{bc}	93.0±4.64^{b}
Group F	106.6±8.28	78.6±3.52^{d}	82.4±8.03^{abc}	90.4±2.66^{cde}	93.4±4.64^{b}
Group G	106.2±8.28	114.2±3.52^{ce}	116.2±8.03^{c}	103.4±2.66^{ef}	101±4.64^{bc}
Group H	116±8.28	113.8±3.52^{ce}	106.4±8.03^{abc}	104.4±2.66^{f}	118.6±4.64^{a}
Group I	115.4±8.28	99.6±3.52^{ae}	111.2±8.03^{ac}	116.6±2.66^{af}	118.8±4.64^{a}

Values are given as mean±SE for 5 rats in each interval in each group (n=20).
Each value not sharing a common letter superscript is significantly different (P<0.05).
* Mean values differ significantly within the group (P<0.05).

Table 4: Effect of different treatment protocols on the aspartate aminotransferase (AST) of BDL rats at various time intervals.

	Pre value	3rd week	4th week	5th week	6th week
Group A	156.79±3.57	156.97±9.8^{a}	158.89±9.8^{a}	156.31±17.25^{a}	157.76±15.56^{a}
Group B	158.05±3.57	268.82±9.8^{b*}	259.46±9.8^{b*}	400.87±17.25^{b*}	630.53±15.56^{b*}
Group C	155.94±3.57	197.83±9.8^{ac*}	191.97±9.8^{ac*}	294.77±17.25^{c*}	312.26±15.56^{d*}
Group D	157.91±3.57	197.47±9.8^{cd*}	211.41±9.8^{c*}	345.66±17.25^{bc*}	436.67±15.56^{c*}
Group E	149.44±3.57	218.97±9.8^{c*}	204.85±9.8^{ac*}	336.62±17.25^{bc*}	439.37±15.56^{c*}
Group F	152.86±3.57	211.13±9.8^{c*}	198.73±9.8^{ac*}	266.31±17.25^{cd*}	329.76±56^{d*}
Group G	151.71±3.57	188.58±9.8^{ac*}	210.86±9.8^{c*}	217.66±17.25^{ad*}	288.76±15.56^{d*}
Group H	148.83±3.57	177.44±9.8^{ac}	190.01±9.8^{ac*}	206.04±17.25^{ad*}	261.67±15.56^{de*}
Group I	151.91±3.57	172.49±9.8^{ac}	182.27±9.8^{ac}	196.19±17.25^{ad*}	227.51±15.56^{ae}

Values are given as mean±SE for 5 rats in each interval in each group (n=20).
Each value not sharing a common letter superscript is significantly different (P<0.05).
* Mean values differ significantly within the group (P<0.05).

Table 5: Effect of different treatment protocols on the alanine aminotransferase (ALT) of BDL rats at various time intervals.

	Pre value	3rd week	4th week	5th week	6th week
Group A	36.49±0.74	39.35±0.73^{a}	40.90±1.17^{a*}	40.46±2.09^{a}	41.21±1.29^{a*}
Group B	38.57±0.74	55.56±0.73^{b*}	96.44±1.17^{b*}	121.41±2.09^{b*}	155.44±1.29^{b*}
Group C	40.08±0.74	49.29±0.73^{cde*}	89.43±1.17^{c*}	97.64±2.09^{cdf*}	130.52±1.29^{df*}
Group D	39.10±0.74	52.26±0.73^{bcd*}	90.97±1.17^{bc*}	106.10±2.09^{c*}	140.66±1.29^{c*}
Group E	38.73±0.74	52.94±0.73^{bd*}	91.21±1.17^{bc*}	103.70±2.09^{cd*}	139.56±1.29^{c*}
Group F	38.94±0.74	50.51±0.73^{de*}	90.42±1.17^{c*}	99.95±2.0^{cdf*}	134.68±1.29^{dc*}
Group G	38.75±0.74	50.29±0.73^{de*}	89.86±1.17^{c*}	95.56±2.09^{def*}	128.71±1.29^{df*}
Group H	39.80±0.74	49.69±0.73^{de*}	88.27±1.17^{c*}	96.07±2.09^{cef*}	125.98±1.29^{ef*}
Group I	39.32±0.74	47.07±0.73^{e*}	86.17±1.17^{c*}	91.78±2.09^{f*}	116.91±1.29^{e*}

Values are given as mean±SE for 5 rats in each interval in each group (n=20).
Each value not sharing a common letter superscript is significantly different (P<0.05).
* Mean values differ significantly within the group (P<0.05).

Table 6: Effect of different treatment protocols on the serum total protein of BDL rats at various time intervals.

	Pre value	3rd week	4th week	5th week	6th week
Group A	6.4±0.18	6.03±0.11^{a*}	6.38±0.1^{a*}	6.21±0.08^{a*}	6.4±0.13^{a*}
Group B	6.38±0.18	4.77±0.11^{b*}	4.58±0.1^{b*}	4.36±0.08^{b*}	3.33±0.13^{b*}
Group C	6.29±0.18	5.39±0.11^{c*}	5.46±0.1^{c*}	5.38±0.08^{cd*}	5.38±0.13^{def*}
Group D	6.33±0.18	5.5±0.11^{cd*}	5.41±0.1^{c*}	5.02±0.08^{c*}	4.62±0.13^{ce*}
Group E	6.45±0.18	5.36±0.11^{c*}	5.43±0.1^{c*}	4.47±0.08^{b*}	4.55±0.13^{c*}
Group F	6.5±0.18	5.36±0.11^{c*}	5.49±0.1^{c*}	5.38±0.08^{cd*}	5.23±0.13^{ef*}
Group G	6.52±0.18	5.53±0.11^{ac*}	5.63±0.1^{c*}	5.59±0.08^{de*}	5.44±0.13^{df*}
Group H	6.37±0.18	5.69±0.11^{ac*}	5.7±0.1^{c*}	5.72±0.08^{de*}	5.64±0.13^{df*}
Group I	6.34±0.18	5.98±0.11^{ad}	5.76±0.1^{c*}	5.9±0.08^{ae}	5.88±0.13^{d}

Values are given as mean±SE for 5 rats in each interval in each group (n=20).
Each value not sharing a common letter superscript is significantly different (P<0.05).
* Mean values differ significantly within the group (P<0.05).

Table 7: Effect of different treatment protocols on the serum albumin of BDL rats at various time intervals.

	Pre value	3rd week	4th week	5th week	6th week
Group A	4.36±0.24	4.4±0.31	4.31±0.44	4.22±0.39	4.37±0.39[a]
Group B	4.29±0.24	3.31±0.31	2.98±0.44	2.46±0.39[a*]	1.98±0.39[b*]
Group C	4.64±0.24	3.37±0.31[*]	3.89±0.44	3.24±0.39[*]	2.81±0.39[*]
Group D	4.32±0.24	3.43±0.31	3.79±0.44	3.37±0.39	3.21±0.39
Group E	4.37±0.24	2.95±0.31[a*]	3.98±0.44	3.37±0.39	3.08±0.39
Group F	4.55±0.24	3.18±0.31[*]	3.83±0.44	3.06±0.39[*]	4.29±0.39[a]
Group G	4.53±0.24	4.07±0.31	3.82±0.44	4.53±0.39[b]	3.98±0.39[a]
Group H	4.38±0.24	4.65±0.31[b]	4.48±0.44	3.88±0.39	4.08±0.39[a]
Group I	4.49±0.24	3.05±0.31[a*]	3.35±0.44	4.09±0.39	3.64±0.39

Values are given as mean±SE for 5 rats in each interval in each group (n=20).
Each value not sharing a common letter superscript is significantly different (P<0.05).
* Mean values differ significantly within the group (P<0.05).

Table 8: Effect of different treatment protocols on the serum globulin of BDL rats at various time intervals.

	Pre value	3rd week	4th week	5th week	6th week
Group A	2.04±0.29	1.64±0.32	2.07±0.45	1.98±0.38	2.04±0.43
Group B	2.09±0.29	1.46±0.32	1.59±0.45	1.9±0.38	1.34±0.43
Group C	2.02±0.29	2.07±0.32	1.62±0.45	1.65±0.38	1.41±0.43
Group D	2.08±0.29	2.41±0.32	1.46±0.45	1.1±0.38	1.47±0.43
Group E	1.65±0.29	2.02±0.32	1.57±0.45	2.14±0.38	2.58±0.43
Group F	1.94±0.29	2.18±0.32	1.66±0.45	2.33±0.38	0.94±0.43
Group G	1.99±0.29	1.46±0.32	1.8±0.45	1.06±0.38	1.46±0.43
Group H	1.99±0.29	1.04±0.32[a]	1.22±0.45	1.84±0.38	1.56±0.43
Group I	1.85±0.29	2.93±0.32[b]	2.41±0.45	1.81±0.38	2.24±0.43

Values are given as mean±SE for 5 rats in each interval in each group (n=20).
Each value not sharing a common letter superscript is significantly different (P<0.05).
* Mean values differ significantly within the group (P<0.05).

Table 9: Effect of different treatment protocols on the alkaline phosphatase of BDL rats at various time intervals.

	Pre value	3rd week	4th week	5th week	6th week
Group A	143.3±6.91	149.65±15.02[a]	147.24±5.7[a]	149.77±6.74[a]	151.34±4.98[a]
Group B	138.61±6.91	235.62±15.02[b*]	253.74±5.7[b*]	271.28±6.74[b*]	295.85±4.98[b*]
Group C	142.52±6.91	218.01±15.02[ab*]	199.74±5.7[c*]	187.41±6.74[ce*]	186.45±4.98[d*]
Group D	146.81±6.91	220.84±15.02[ab*]	244.45±5.7[b*]	251.41±6.74[b*]	238.48±4.98[c*]
Group E	143.90±6.91	227.36±15.02[ab*]	243.57±5.7[b*]	219.00±6.74[bd*]	228.25±4.98[c*]
Group F	141.55±6.91	194.64±15.02[ab*]	199.95±5.7[c*]	193.67±6.74[cde*]	176.19±4.98[d*]
Group G	139.25±6.91	205.80±15.02[ab*]	199.21±5.7[c*]	167.54±6.74[ae*]	171.57±4.98[d*]
Group H	140.64±6.91	160.48±15.02[a]	198.70±5.7[c*]	182.53±6.74[ae*]	167.21±4.98[d*]
Group I	142.09±6.91	181.72±15.02[ab]	175.09±5.7[c*]	175.37±6.74[ae*]	164.88±4.98[d*]

Values are given as mean±SE for 5 rats in each interval in each group (n=20).
Each value not sharing a common letter superscript is significantly different (P<0.05).
* Mean values differ significantly within the group (P<0.05).

Table 10: Effect of different treatment protocols on the gamma glutamyl transferase of BDL rats at various time intervals.

	Pre value	3rd week	4th week	5th week	6th week
Group A	4.49±0.19	4.75±0.73[a]	4.5±0.64[a]	4.18±1.83[a]	5.28±1.12[a]
Group B	4.29±0.19	24.30±0.73[b*]	46.56±0.64[b*]	74.08±1.83[b*]	116.42±1.12[b*]
Group C	4.73±0.19	19.20±0.73[c*]	38.15±0.64[d*]	59.96±1.83[cd*]	104.45±1.12[cd*]
Group D	4.32±0.19	24.19±0.73[cd*]	45.77±0.64[bh*]	67.4±1.83[bc*]	108.89±1.12[bc*]
Group E	3.83±0.19[a]	22.66±0.73[c*]	42.53±0.64[ce*]	67.92±1.83[bc*]	111.23±1.12[b*]
Group F	4.28±0.19	20.82±0.73[c*]	42.71±0.64[eh*]	56.33±1.83[d*]	99.06±1.12[d*]
Group G	4.8±0.19[b]	17.23±0.73[ac*]	34.49±0.64[f*]	49.55±1.83[e*]	82.68±1.12[e*]
Group H	4.59±0.19	19.73±0.73[ac*]	34.33±0.64[f*]	50.73±1.83[e*]	82.34±1.12[e*]
Group I	4.26±0.19	14.4±0.73[ad*]	25.2±0.64[g*]	36.76±1.83[f*]	72.01±1.12[f*]

Values are given as mean±SE for 5 rats in each interval in each group (n=20).
Each value not sharing a common letter superscript is significantly different (P<0.05).
* Mean values differ significantly within the group (P<0.05).

Table 11: Effect of different treatment protocols on the total bilirubin of BDL rats at various time intervals

	Pre value	3rd week	4th week	5th week	6th week
Group A	0.43±0.03	0.45±0.08[a]	0.41±0.14[ac]	0.41±0.09[a]	0.41±0.12[a]
Group B	0.48±0.03	1.31±0.08[b*]	1.49±0.14[b*]	2.26±0.09[b*]	2.54±0.12[b*]
Group C	0.37±0.03	0.57±0.08[a]	0.91±0.14[abc*]	1.46±0.09[cd*]	1.42±0.12[cd*]
Group D	0.45±0.03	0.74±0.08[a*]	1.28±0.14[a*]	1.58±0.09[c*]	1.83±0.12[c*]
Group E	0.46±0.03	0.76±0.08[a*]	0.95±0.14[ac*]	1.04±0.09[de*]	1.81±0.12[c*]
Group F	0.43±0.03	0.53±0.08[a]	1.26±0.14[abc*]	1.32±0.09[cd*]	1.64±0.12[c*]
Group G	0.36±0.03	0.55±0.08[a]	1.13±0.14[abc*]	1.24±0.09[cd*]	1.27±0.12[cde*]
Group H	0.39±0.03	0.4±0.08[a]	0.61±0.14[abc*]	1.24±0.09[cd*]	1.04±0.12[de*]
Group I	0.40±0.03	0.42±0.08[a]	0.6±0.14[c]	0.77±0.09[e*]	0.77±0.12[ae*]

Values are given as mean±SE for 5 rats in each interval in each group (n=20).
Each value not sharing a common letter superscript is significantly different (P<0.05).
* Mean values differ significantly within the group (P<0.05).

Table 12: Effect of different treatment protocols on the direct bilirubin of BDL rats at various time intervals.

	Pre value	3rd week	4th week	5th week	6th week
Group A	0.17±0.03	0.14±0.11	0.13±0.11[a]	0.18±0.14	0.2±0.12[a]
Group B	0.18±0.03	0.58±0.11[*]	1.1±0.11[b*]	0.85±0.14[*]	1.81±0.12[b*]
Group C	0.22±0.03	0.17±0.11	0.31±0.11[a]	0.71±0.14	0.15±0.12[a]
Group D	0.2±0.03	0.22±0.11	0.66±0.11[ab*]	0.35±0.14	0.59±0.12[a*]
Group E	0.29±0.03	0.42±0.11	0.27±0.11[a]	0.29±0.14	0.53±0.12[a]
Group F	0.27±0.03	0.28±0.11	0.22±0.11[a]	0.19±0.14	0.24±0.12[a]
Group G	0.19±0.03	0.20±0.11	0.42±0.11[a]	0.41±0.14	0.33±0.12[a]
Group H	0.28±0.03	0.42±0.11	0.26±0.11[a]	0.34±0.14	0.23±0.12[a]
Group I	0.21±0.03	0.23±0.11	0.26±0.11[a]	0.29±0.14	0.28±0.12[a]

Values are given as mean±SE for 5 rats in each interval in each group (n=20).
Each value not sharing a common letter superscript is significantly different (P<0.05).
* Mean values differ significantly within the group (P<0.05).

Table 13: Effect of different treatment protocols on the indirect bilirubin of BDL rats at various time intervals.

	Pre value	3rd week	4th week	5th week	6th week
Group A	0.26±0.04	0.31±0.11^{ab}	0.28±0.15^{a}	0.23±0.19^{c}	0.21±0.18^{a}
Group B	0.3±0.04	0.74±0.11^{a*}	0.38±0.15^{ab}	1.41±0.19^{a*}	0.73±0.18^{ab}
Group C	0.15±0.04	0.41±0.11^{ab}	0.59±0.15^{ab*}	0.75±0.19^{b*}	1.27±0.18^{bc*}
Group D	0.25±0.04	0.52±0.11^{a}	0.62±0.15^{ab*}	1.23±0.19^{b*}	1.24±0.18^{bc*}
Group E	0.17±0.04	0.34±0.11^{ab}	0.68±0.15^{ab*}	0.75±0.19^{b*}	1.28±0.18^{bc}
Group F	0.16±0.04	0.25±0.11^{ab}	1.04±0.15^{ab*}	1.13±0.19^{b*}	1.41±0.18^{b*}
Group G	0.17±0.04	0.35±0.11ab	0.71±0.15^{b*}	0.83±0.19^{bc*}	0.94±0.18^{abc*}
Group H	0.11±0.04	0.14±0.11b	0.35±0.15^{ab*}	0.9±0.19^{bc*}	0.82±0.18^{abc*}
Group I	0.19±0.04	0.18±0.11b	0.34±0.15^{ab*}	0.47±0.19^{b*}	0.49±0.18^{ac*}

Values are given as mean±SE for 5 rats in each interval in each group (n=20).
Each value not sharing a common letter superscript is significantly different (P<0.05).
* Mean values differ significantly within the group (P<0.05).

Table 14: Effect of different treatment protocols on the total lipids of BDL rats at various time intervals.

	Pre value	3rd week	4th week	5th week	6th week
Group A	155.78±0.55	156.23±3.7^{a}	157.6±2.98^{a}	155.56±6.12^{a}	155.75±4.7^{a}
Group B	156.04±0.55	302.91±3.7^{b*}	600.86±2.98^{b*}	638.16±6.12^{b*}	708.83±4.7^{b*}
Group C	156.33±0.55	265.74±3.7^{cd*}	451.6±2.98^{e*}	549.53±6.12^{e*}	621.53±4.7^{e*}
Group D	154.11±0.55	288.15±3.7^{b*}	505.35±2.98^{c*}	577.87±6.12^{c*}	676.27±4.7^{c*}
Group E	156.1±0.55	291.42±3.7^{b*}	491.62±2.98^{cd*}	588.61±6.12^{cd*}	653.6±4.7^{cd*}
Group F	155.65±0.55	248.34±3.7^{d*}	455.17±2.98^{e*}	538.7±6.12^{e*}	612.83±4.7^{e*}
Group G	154.26±0.55	225.28±3.7^{e*}	421.82±2.98^{f*}	535.04±6.12^{e*}	601.33±4.7^{e*}
Group H	156.26±0.55	223.85±3.7^{e*}	416.74±2.98^{f*}	531.08±6.12^{f*}	570.84±4.7^{f*}
Group I	155.66±0.55	217.09±3.7^{e*}	388.69±2.98^{h*}	369.4±6.12^{g*}	392.43±4.7^{g*}

Values are given as mean±SE for 5 rats in each interval in each group (n=20).
Each value not sharing a common letter superscript is significantly different (P<0.05).
* Mean values differ significantly within the group (P<0.05).

Table 15: Effect of different treatment protocols on the triglycerides of BDL rats at various time intervals.

	Pre value	3rd week	4th week	5th week	6th week
Group A	73.67±0.41^{a}	75.16±0.95^{a}	74.63±0.44^{a}	74.76±0.97^{a}	74.89±0.6^{a}
Group B	74.77±0.41	86.16±0.95^{b*}	97.96±0.44^{b*}	101.65±0.97^{b*}	117.13±0.6^{b*}
Group C	75.48±0.41	80.37±0.95^{bde*}	91.62±0.44^{d*}	91.81±0.97^{c*}	99.95±0.6^{d*}
Group D	74.81±0.41	81.37±0.95^{bc*}	95.15±0.44^{c*}	93.60±0.97^{c*}	109.21±0.6^{c*}
Group E	76±0.41^{b}	83.55±0.95^{cdf*}	94.97±0.44^{c*}	95.19±0.97^{c*}	106.56±0.6^{c*}
Group F	74.83±0.41	83.54±0.95^{bde*}	92.67±0.44^{d*}	93.54±0.97^{c*}	99.07±0.6^{d}
Group G	75.1±0.41	79.5±0.95^{acdef*}	88.72±0.44^{e*}	91.28±0.97^{cd*}	98.66±0.6^{d}
Group H	74.51±0.41	80.28±0.95^{cdf*}	87.11±0.44^{e*}	91.23±0.97^{cd*}	97.55±0.6^{d*}
Group I	75.81±0.45^{b}	78.48±1.06^{adf}	83.10±0.49^{f*}	86.38±1.08^{d*}	90.34±0.68^{e*}

Values are given as mean±SE for 5 rats in each interval in each group (n=20).
Each value not sharing a common letter superscript is significantly different (P<0.05).
* Mean values differ significantly within the group (P<0.05).

Table 16: Effect of different treatment protocols on the high-density lipoprotein (HDL) of BDL rats at various time intervals.

	Pre value	3rd week	4th week	5th week	6th week
Group A	43.54±0.47	44.92±0.73^{a}	43.65±0.49^{a}	43.2±0.41^{a}	44.3±0.44^{a}
Group B	43.92±0.47	34.26±0.73^{b*}	28.18±0.49^{b*}	23.66±0.41^{b*}	18.59±0.44^{b*}
Group C	43.38±0.47	33.75±0.73^{b*}	38.14±0.49^{c*}	40.19±0.41^{c*}	41.47±0.44^{cd*}
Group D	42.36±0.47	31.51±0.73^{b*}	35.88±0.49$^{c`*}$	37.64±0.41^{d*}	39.66±0.44^{c*}
Group E	42.38±0.47	36.08±0.73^{b*}	35.93±0.49^{c*}	37.10±0.41^{d*}	39.77±0.44^{c*}
Group F	43.16±0.47	39.92±0.73^{c*}	37.5±0.49^{c*}	38.61±0.41^{cd*}	42.11±0.44^{d*}
Group G	41.96±0.47	43.16±0.73^{a}	45.62±0.49^{ad*}	48.15±0.41^{e*}	51.92±0.44^{e*}
Group H	42.05±0.47	45.11±0.73^{a*}	47.23±0.49^{de*}	48.78±0.41^{e*}	52.87±0.44^{e*}
Group I	42.52±0.47	48.39±0.73^{a*}	48.67±0.49^{e*}	53.24±0.41^{f*}	59.68±0.44^{f*}

Values are given as mean±SE for 5 rats in each interval in each group (n=20).
Each value not sharing a common letter superscript is significantly different (P<0.05).
* Mean values differ significantly within the group (P<0.05).

Table 17: Effect of different treatment protocols on the low-density lipoprotein (LDL) of BDL rats at various time intervals.

	Pre value	3rd week	4th week	5th week	6th week
Group A	23.61±0.32	23.9±0.35[a]	24.45±0.48[a]	24.52±0.59[a]	24.89±0.51[a]
Group B	24.11±0.32	36.77±0.35[b*]	42.96±0.48[b*]	58.62±0.59[b*]	74.53±0.51[b*]
Group C	24.68±0.32	31.45±0.35[d*]	40.86±0.48[bc*]	55.01±0.59[c*]	66.19±0.51[d*]
Group D	24.3±0.32	34.8±0.35[c*]	42.67±0.48[b*]	56.8±0.59[bc*]	71.51±0.51[c*]
Group E	24.52±0.32	33.8±0.35[c*]	41.06±0.48[bc*]	56.56±0.59[bc*]	71.67±0.51[c*]
Group F	24.18±0.32	30.9±0.35[d*]	38.87±0.48[cd*]	51.11±0.59[d*]	62.67±0.51[e*]
Group G	24.64±0.32	30.13±0.35[d*]	40.1±0.48[cd*]	47.37±0.59[e*]	48.81±0.51[f*]
Group H	24.92±0.32	30.5±0.35[d*]	38.31±0.48[d*]	47.99±0.59[e*]	48.21±0.51[f*]
Group I	24.72±0.32	27.67±0.35[e*]	31.04±0.48[e*]	37.91±0.59[f*]	40.42±0.51[g*]

Values are given as mean±SE for 5 rats in each interval in each group (n=20).
Each value not sharing a common letter superscript is significantly different (P<0.05).
* Mean values differ significantly within the group (P<0.05).

Table 18: Effect of different treatment protocols on the total cholesterol of BDL rats at various time intervals

	Pre value	3rd week	4th week	5th week	6th week
Group A	53.62±0.46	53.60±0.71[a]	53.13±0.49[a]	52.69±0.43[a]	53.79±0.44[a]
Group B	53.40±0.46	88.56±0.71[b*]	95.49±0.49[b*]	101.96±0.43[b*]	117.69±0.44[b*]
Group C	52.86±0.46	76.84±0.71[c*]	80.48±0.49[c*]	74.29±0.43[ce*]	72.45±0.44[ce*]
Group D	52.25±0.46	79.56±0.71[ce*]	82.93±0.49[d*]	76.73±0.43[d*]	76.65±0.44[d*]
Group E	51.87±0.46	84.13±0.71[d*]	77.39±0.49[e*]	76.19±0.43[cd*]	76.76±0.44[d*]
Group F	52.65±0.46	83.01±0.71[de*]	78.9±0.49[ce*]	72.71±0.43[eg*]	73.10±0.44[e*]
Group G	51.44±0.46	70.57±0.71[f*]	69.72±0.49[fg*]	70.24±0.43[f*]	64.10±0.44[f*]
Group H	51.53±0.46	72.51±0.71[f*]	71.33±0.49[f]	70.87±0.43[fg*]	65.05±0.44[f]
Group I	52.00±0.46	70.59±0.71[f*]	67.99±0.49[g*]	65.74±0.43[h*]	61.88±0.44[h*]

Values are given as mean±SE for 5 rats in each interval in each group (n=20).
Each value not sharing a common letter superscript is significantly different (P<0.05).
* Mean values differ significantly within the group (P<0.05).

Table 19: Effect of different treatment protocols on the serum creatinine of BDL rats at various time intervals.

	Pre value	3rd week	4th week	5th week	6th week
Group A	1.26±0.03	1.28±0.04^{ab}	1.28±0.03^{a}	1.21±0.07^{a}	1.27±0.09^{a}
Group B	1.24±0.03	1.43±0.04^{a}	1.60±0.03^{b*}	1.86±0.07^{b*}	2.77±0.09^{b*}
Group C	1.23±0.03	1.27±0.04^{ab}	1.28±0.03^{a*}	1.38±0.07^{a*}	1.33±0.09^{a*}
Group D	1.29±0.03	1.28±0.04^{ab}	1.39±0.03^{a}	1.46±0.07^{a}	1.42±0.09^{a}
Group E	1.28±0.03	1.29±0.04^{ab}	1.27±0.03^{a}	1.45±0.07^{a}	1.44±0.09^{a}
Group F	1.29±0.03	1.24±0.04^{b}	1.33±0.03^{a}	1.34±0.07^{a}	1.40±0.09^{a}
Group G	1.30±0.03	1.19±0.04^{b}	1.28±0.03^{a}	1.32±0.07^{a}	1.31±0.09^{a}
Group H	1.25±0.03	1.17±0.04^{b}	1.33±0.03^{a}	1.38±0.07^{a}	1.30±0.09^{a}
Group I	1.27±0.03	1.15±0.04^{b}	1.39±0.03^{a*}	1.35±0.07^{a*}	1.25±0.09^{a}

Values are given as mean±SE for 5 rats in each interval in each group (n=20).
Each value not sharing a common letter superscript is significantly different (P<0.05).
* Mean values differ significantly within the group (P<0.05).

Table 20: Effect of different treatment protocols on the serum urea of BDL rats at various time intervals.

	Pre value	3rd week	4th week	5th week	6th week
Group A	19.3±0.32	19.25±0.39^{a}	19.97±0.54^{ac}	19.96±0.41^{a}	20.62±0.3^{a}
Group B	20.1±0.32	23.28±0.39^{b**}	27.2±0.54^{b**}	30.52±0.41^{b**}	30.87±0.3^{b**}
Group C	20.41±0.32	21.36±0.39^{bc**}	23.61±0.54^{b**}	27.83±0.41^{b**}	29.02±0.3^{b**}
Group D	20±0.32	23.2±0.39^{bc**}	27.21±0.54^{bd**}	31.09±0.41^{be**}	30.91±0.3^{bc**}
Group E	20.07±0.32	22.69±0.39^{c}	24.86±0.54^{cde**}	30.06±0.41^{cd**}	29.72±0.3^{ce**}
Group F	20.46±0.32	22.84±0.39^{bc**}	24.75±0.54^{bcd**}	29.45±0.41^{bc**}	30.23±0.3^{bc**}
Group G	19.81±0.32	21.63±0.39^{bc**}	23.48±0.54^{de**}	26.89±0.41^{d**}	26.41±0.3^{d**}
Group H	20.06±0.32	21.71±0.39^{bc**}	23.46±0.54^{de**}	28.18±0.41^{cde**}	28.13±0.3^{e**}
Group I	20.06±0.32	21.46±0.39^{bc}	22.02±0.54^{e*}	22.8±0.41^{f**}	24.66±0.3^{f**}

Values are given as mean±SE for 5 rats in each interval in each group (n=20).
Each value not sharing a common letter superscript is significantly different (P<0.05).
* Mean values differ significantly within the group (P<0.05).

Table 21: Effect of different treatment protocols on the blood urea nitrogen (BUN) of BDL rats at various time intervals.

Values are given as mean±SE for 5 rats in each interval in each group (n=20).

	Pre value	3rd week	4th week	5th week	6th week
Group A	38.39±0.79^{ac}	42.57±0.82^{ad*}	40.96±0.65^{a}	40.7±0.87^{a}	41.28±1.15^{a}
Group B	44.02±0.79^{b}	40.12±0.82^{ad*}	35.83±0.65^{b}	35.92±0.87^{b*}	30.36±1.15^{b*}
Group C	41.27±0.79^{ab}	41.48±0.82^{ab}	41.87±0.65^{a}	44.09±0.87^{a*}	45.51±1.15^{a}
Group D	40.54±0.79^{abc}	38.75±0.82^{a}	42.27±0.65^{a}	41.47±0.87^{a}	40.52±1.15^{a}
Group E	39.14±0.79^{ac}	42.97±0.82^{bc*}	48.62±0.65^{c*}	52.71±0.87^{c*}	51.36±1.15^{c*}
Group F	38.68±0.79^{abc}	46.81±0.82^{c*}	48.95±0.65^{c*}	54.67±0.87^{cd*}	53.47±1.15^{cd*}
Group G	37.3±0.79^{c}	41.8±0.82^{ab*}	48.39±0.65^{c*}	57.74±0.87^{de*}	55.23±1.15^{cd*}
Group H	38.31±0.79^{ac}	43.29±0.82^{bcd*}	54.28±0.65^{d*}	60.55±0.87^{e*}	57.38±1.15^{de*}
Group I	38.54±0.79^{ac}	44.4±0.82^{bc*}	54.8±0.65^{d*}	61.38±0.87^{e*}	62.66±1.15^{e*}

Each value not sharing a common letter superscript is significantly different (P<0.05).
* Mean values differ significantly within the group (P<0.05).

Table 22: Effect of different treatment protocols on the serum hepatocyte growth factor level of BDL rats at various time intervals.

	Pre value	3rd week	4th week	5th week	6th week
Group A	9.6±0.46	9.58±0.52^{a}	9.11±0.35^{a}	8.67±0.35^{a}	9.77±0.36^{a}
Group B	9.38±0.46	15.49±0.52^{b*}	17.69±0.35^{b*}	20.89±0.35^{b**}	27.21±0.36^{b*}
Group C	8.84±0.46	9.88±0.52^{a}	11.08±0.35^{ac*}	13.18±0.35^{ce*}	15.22±0.36^{ce*}
Group D	8.23±0.46	9.77±0.52^{a*}	12.87±0.35^{d*}	14.97±0.35^{d*}	17.00±0.36^{d*}
Group E	7.85±0.46	9.39±0.52^{a*}	12.49±0.35^{d*}	14.58±0.35^{cd*}	16.62±0.36^{cd*}
Group F	8.63±0.46	9.67±0.52^{a}	10.87±0.35^{ac*}	12.59±0.35^{e*}	14.62±0.36^{e*}
Group G	7.42±0.46	8.18±0.52^{a}	10.28±0.35^{a**}	11.53±0.35^{ae*}	13.63±0.36^{eg*}
Group H	7.52±0.46	8.73±0.52^{a}	10.34±0.35^{a*}	11.60±0.35^{ae*}	13.70±0.36^{eg*}
Group I	7.98±0.46	8.25±0.52^{a}	9.37±0.35^{a*}	10.2±0.35^{a*}	12.30±0.36^{g*}

Values are given as mean±SE for 5 rats in each interval in each group (n=20).
Each value not sharing a common letter superscript is significantly different (P<0.05).
* Mean values differ significantly within the group (P<0.05).

Table 23: Effect of different treatment protocols on the MMP-2 of BDL rats at various time intervals.

	Pre value	3rd week	4th week	5th week	6th week
Group A	6.83±0.16	6.76±1.92[a]	7.67±2.71[a]	8.34±3.08[a]	9.48±3.43[a]
Group B	6.72±0.16	80.75±1.92[b*]	75.8±2.71[b*]	84.05±3.08[b*]	125.11±3.43[b*]
Group C	6.89±0.16	80.17±1.92[b*]	94.37±2.71[cd*]	129.69±3.08[cd*]	185.22±3.43[b*]
Group D	7.2±0.16	78.95±1.92[b*]	91.28±2.71[cd*]	122.38±3.08[c*]	183.76±3.43[cd*]
Group E	6.93±0.16	79.97±1.92[b*]	82.58±2.71[bc*]	129.63±3.08[cd*]	175.87±3.43[cd*]
Group F	6.86±0.16	82.46±1.92[b*]	96.84±2.71[d*]	127.65±3.08[cd*]	185.88±3.43[c*]
Group G	7.32±0.16	82.58±1.92[b*]	96.68±2.71[d*]	133.17±3.08[cd*]	189.61±3.43[cd*]
Group H	7.63±0.16	83.58±1.92[b*]	101.94±2.71[d*]	141.62±3.08[de*]	189.73±3.43[cd*]
Group I	6.85±0.16	88.19±1.92[b*]	116.28±2.71[e*]	156.43±3.08[e*]	195.22±3.43[d*]

Values are given as mean±SE for 5 rats in each interval in each group (n=20).
Each value not sharing a common letter superscript is significantly different (P<0.05).
* Mean values differ significantly within the group (P<0.05).

Table 24: Effect of different treatment protocols on the TIMP-2 of BDL rats at various time intervals.

	Pre value	3rd week	4th week	5th week	6th week
Group A	4.53±0.16[a]	4.45±0.42[a]	5.36±0.77[a]	6.03±1.07[a]	7.18±2.09[a]
Group B	4.41±0.16[b]	13.45±0.42[b*]	21.44±0.77[b*]	37.75±1.07[b*]	78.8±2.09[b*]
Group C	4.58±0.16	10.91±0.42[ce*]	17.51±0.77[c*]	23.68±1.07[cd*]	42.77±2.09[c*]
Group D	4.89±0.16	11.64±0.42[bcd*]	19.44±0.77[bc*]	26.12±1.07[c*]	41.5±2.09[c*]
Group E	4.63±0.16	12.66±0.42[bc*]	18.93±0.77[bc*]	25.83±1.07[c*]	46.14±2.09[c*]
Group F	4.56±0.16	11.3±0.42[cd*]	18.66±0.77[c*]	23.65±1.07[cd*]	42.14±2.09[c*]
Group G	5.02±0.16	10.07±0.42[d*]	18.2±0.77[bc*]	23.55±1.07[cd*]	38.7±2.09[c*]
Group H	5.33±0.16[b]	10.63±0.42[ed*]	18.65±0.77[bc*]	24.05±1.07[cd*]	35.94±2.09[cd*]
Group I	4.54±0.16[a]	9.15±0.42[ef*]	13.4±0.77[d*]	19.52±1.07[d*]	27.73±2.09[d*]

Values are given as mean±SE for 5 rats in each interval in each group (n=20).
Each value not sharing a common letter superscript is significantly different (P<0.05).
* Mean values differ significantly within the group (P<0.05).

Table 25: Effect of different treatment protocols on the total antioxidant capacity (TAC) of BDL rats at various time intervals.

	Pre value	3rd week	4th week	5th week	6th week
Group A	14.67±1.48	15.41±2.1[a]	8.07±1.61[a]	13.15±1.08[a]	12.49±1.4[a]
Group B	12.51±1.48	45.56±2.1[b*]	51.31±1.61[b*]	45.56±1.08[b*]	40.21±1.4[b*]
Group C	10.25±1.48	50.64±2.1[b*]	50.05±1.61[b*]	63.91±1.08[c*]	66.96±1.4[c*]
Group D	11.91±1.48	49.46±2.1[b*]	54.38±1.61[bd*]	64.84±1.08[c*]	64.93±1.4[c*]
Group E	13.36±1.48	53.93±2.1[bc*]	64.06±1.61[c*]	67.00±1.08[c*]	69.82±1.4[c*]
Group F	13.72±1.48	56.35±2.1[bc*]	61.33±1.61[cd*]	65.89±1.08[c*]	71.09±1.4[c*]
Group G	9.5±1.48	61.46±2.1[c*]	76.5±1.61[e*]	84.5±1.08[d*]	86.09±1.4[d*]
Group H	10.99±1.48	61.66±2.1[c*]	79.81±1.61[ef*]	94.66±1.08[e*]	89.01±1.4[e*]
Group I	11.48±1.48	63.45±2.1[c*]	87.16±1.61[f*]	99.14±1.08[e*]	112.43±1.4[e*]

Values are given as mean±SE for 5 rats in each interval in each group (n=20).
Each value not sharing a common letter superscript is significantly different (P<0.05).
* Mean values differ significantly within the group (P<0.05).

Table 26: Effect of different treatment protocols on the superoxide dismutase (SOD) of BDL rats at various time intervals.

	Pre value	3rd week	4th week	5th week	6th week
Group A	10.67±1.48	11.41±2.1[a]	04.07±1.61[a]	9.15±1.08[a]	8.49±1.4[a]
Group B	9.51±1.48	22.06±2.1[b]	27.81±1.61[b]	22.06±1.08[b]	16.71±1.4[b]
Group C	7.25±1.48	27.14±2.1[bc]	26.55±1.61[b]	40.41±1.08[c]	43.46±1.4[c]
Group D	8.91±1.48	25.96±2.1[bc]	30.88±1.61[b]	41.34±1.08[c]	41.43±1.4[c]
Group E	10.36±1.48	30.43±2.1[bcd]	40.56±1.61[c]	43.5±1.08[c]	46.32±1.4[c]
Group F	10.72±1.48	32.85±2.1[cd]	37.83±1.61[c]	42.39±1.08[c]	47.59±1.4[c]
Group G	6.50±1.48	37.96±2.1[d]	53.00±1.61[d]	61.00±1.08[d]	62.59±1.4[d]
Group H	7.99±1.48	38.16±2.1[d]	56.31±1.61[e]	71.16±1.08[e]	65.51±1.4[e]
Group I	8.48±1.48	39.95±2.1[d]	63.66±1.61[f]	75.64±1.08[e]	88.93±1.4[e]

Values are given as mean±SE for 5 rats in each interval in each group (n=20).
Each value not sharing a common letter superscript is significantly different (P<0.05).
* Mean values differ significantly within the group (P<0.05).

Table 27: Effect of different treatment protocols on the percentage of fibrosis of BDL rats at various time intervals.

	4th week	6th week	Reduction percentage at 4th week (%)	Reduction percentage at 6th week (%)
Group A	1.39 ± 0.39^{a}	4.21 ± 0.53^{a}		
Group B	$9.9 \pm 0.39^{b*}$	$32.43 \pm 0.53^{b*}$		
Group C	$2.55 \pm 0.39^{ac*}$	$14.96 \pm 0.53^{c*}$	74.74	53.86
Group D	$4.42 \pm 0.39^{c*}$	$23.86 \pm 0.53^{d*}$	55.35	26.86
Group E	$3.82 \pm 0.39^{cde*}$	$24.16 \pm 0.53^{d*}$	61.41	25.50
Group F	$2.35 \pm 0.39^{ad*}$	$13.26 \pm 0.53^{c*}$	76.26	59.42
Group G	$2.05 \pm 0.39^{ae*}$	$9.68 \pm 0.53^{e*}$	79.29	70.15
Group H	$1.71 \pm 0.39^{a*}$	$8.68 \pm 0.53^{ef*}$	82.72	73.23
Group I	$1.91 \pm 0.39^{a*}$	$6.58 \pm 0.53^{f*}$	80.70	79.71

Values are given as mean±SE for 5 rats in each interval in each group (n=20).
Each value not sharing a common letter superscript is significantly different (P<0.05).
* Mean values differ significantly within the group (P<0.05).

Table 28: Effects of different treatment protocol on necro-inflammatory and fibrosis scores (mean±SD) in the BDL rats.

**Score**	**HAI score**				**Knodell score**				**METAVIR score**			
**Groups weeks**	**3**	**4**	**5**	**6**	**3**	**4**	**5**	**6**	**3**	**4**	**5**	**6**
Group A	0.2±0.4[a]	0.4±0.5[a]	0.0±0.0[a]	0.2±0.4[a]	0.0±0.0[a]	0.0±0.0[a]	0.0±0.0[a]	0.0±0.0[a]	0.0±0.0[a]	0.0±0.0[a]	0.0±0.0[a]	0.0±0.0[a]
Group B	6.8±0.8[b]	8.6±0.5[b]	10.8±0.8[b*]	12.4±0.5[b*]	1.0±0.0[b]	2.2±1.0[b]	2.6±0.8[b*]	3.8±0.4[b*]	1.0±0.0[b]	1.8±0.4[b*]	2.8±0.4[b*]	3.8±0.4[b*]
Group C	4.2±0.8[c]	5.6±0.5[ce]	7.0±0.7[cg*]	7.6±0.5[c*]	0.8±0.4[b]	0.8±0.4[c]	1.2±1.0[bc]	1.8±1.0[bce]	0.8±0.4[b]	1.4±0.5[b]	1.4±0.5[b]	1.8±0.8[cd]
Group D	5.0±1.5 [c]	6.4±0.8[c]	9.4±0.5[d*]	9.8±0.4[d*]	1.0±0.0[b]	2.2±1.0[b]	2.2±1.0[b]	2.4±1.3[bc]	1.0±0.0[b]	2.0±1.0[b]	1.8±0.8[c]	2.0±1.0[cd]
Group E	5.0±1.2 [c]	6.6±1.1[c]	9.4±0.5[d*]	9.8±0.4[d*]	1.0±0.0[b]	2.6±0.8[bd]	2.6±0.8[b*]	2.2±1.0[c]	1.2±0.4[b]	2.2±0.8[b]	2.6±0.8[bc]	2.4±0.8[c]
Group F	3.8±0.8 [c]	6.0±0.7[cf]	7.4±0.5[c*]	8.0±0.7[c*]	0.8±0.4[b]	1.0±0.0[bc]	1.4±0.8[bd]	1.8±1.0[cd]	0.6±0.5[b]	1.4±0.5[b]	1.4±0.8[bd]	1.6±0.8[cd]
Group G	4.4±0.5 [c]	4.4±0.5[d]	6.2±0.8[eg*]	6.4±0.5[e*]	0.6±0.5[b]	0.8±0.4[ec]	1.0±0.0[cd]	1.4±0.8[cd]	0.6±0.5[b]	0.8±0.4[b]	1.2±0.4[cde]	1.6±0.8[cd]
Group H	4.2±0.8 [c]	5.0±0.7[edf]	5.4±0.5[e]	6.6±0.5[e*]	0.6±0.5[b]	0.8±0.4[ce]	0.8±0.4[cde]	1.4±0.8[cd]	0.6±0.5[b]	1.0±0.7[b]	1.0±0.0[ce]	1.4±0.5[cd]
Group I	4.2±0.4 [c]	4.4±0.5[d]	4.0±0.7[f]	3.8±0.4[f]	0.4±0.5[ab]	0.4±0.5[ac]	0.2±0.4[ae]	0.8±0.4[d]	0.6±0.5[b]	0.6±0.8[b]	0.6±0.5[de]	1.0±0.7[d]
